# Rapalogs downmodulate intrinsic immunity and promote cell entry of SARS-CoV-2

**DOI:** 10.1172/JCI160766

**Published:** 2022-12-15

**Authors:** Guoli Shi, Abhilash I. Chiramel, Tiansheng Li, Kin Kui Lai, Adam D. Kenney, Ashley Zani, Adrian C. Eddy, Saliha Majdoul, Lizhi Zhang, Tirhas Dempsey, Paul A. Beare, Swagata Kar, Jonathan W. Yewdell, Sonja M. Best, Jacob S. Yount, Alex A. Compton

**Affiliations:** 1 HIV Dynamics and Replication Program, Center for Cancer Research, National Cancer Institute (NCI), NIH, Frederick, Maryland, USA.; 2 Laboratory of Virology, Rocky Mountain Laboratories, National Institute of Allergy and Infectious Diseases (NIAID), NIH, Hamilton, Montana, USA.; 3 Laboratory of Viral Diseases, NIAID, NIH, Bethesda, Maryland, USA.; 4 Department of Microbial Infection and Immunity, The Ohio State University, Columbus, Ohio, USA.; 5 Laboratory of Bacteriology, Rocky Mountain Laboratories, NIAID, NIH, Hamilton, Montana, USA.; 6 Bioqual, Rockville, Maryland, USA.

**Keywords:** COVID-19, Autophagy, Innate immunity, Lysosomes

## Abstract

Severe acute respiratory syndrome coronavirus 2 (SARS-CoV-2) infection in immunocompromised individuals is associated with prolonged virus shedding and evolution of viral variants. Rapamycin and its analogs (rapalogs, including everolimus, temsirolimus, and ridaforolimus) are FDA approved as mTOR inhibitors for the treatment of human diseases, including cancer and autoimmunity. Rapalog use is commonly associated with an increased susceptibility to infection, which has been traditionally explained by impaired adaptive immunity. Here, we show that exposure to rapalogs increased susceptibility to SARS-CoV-2 infection in tissue culture and in immunologically naive rodents by antagonizing the cell-intrinsic immune response. We identified 1 rapalog (ridaforolimus) that was less potent in this regard and demonstrated that rapalogs promote spike-mediated entry into cells, by triggering the degradation of the antiviral proteins IFITM2 and IFITM3 via an endolysosomal remodeling program called microautophagy. Rapalogs that increased virus entry inhibited mTOR-mediated phosphorylation of the transcription factor TFEB, which facilitated its nuclear translocation and triggered microautophagy. In rodent models of infection, injection of rapamycin prior to and after virus exposure resulted in elevated SARS-CoV-2 replication and exacerbated viral disease, while ridaforolimus had milder effects. Overall, our findings indicate that preexisting use of certain rapalogs may elevate host susceptibility to SARS-CoV-2 infection and disease by activating lysosome-mediated suppression of intrinsic immunity.

## Graphical abstract

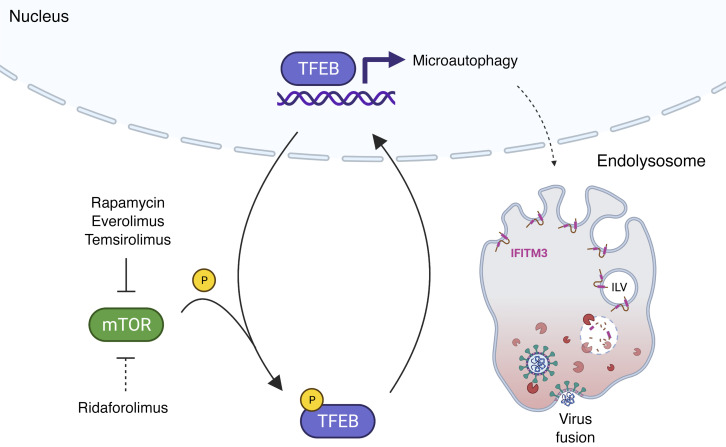

## Introduction

Severe acute respiratory syndrome coronavirus 2 (SARS-CoV-2) emerged in humans in 2019 following a species jump from bats and is the cause of COVID-19, a respiratory and multiorgan disease of variable severity (1, 2). The characterization of virus-host interactions that dictate SARS-CoV-2 infection and COVID-19 severity is a major priority for public health (3). Immune impairment, such as that resulting from cancer, has been associated with prolonged SARS-CoV-2 shedding, the seeding of “super-spreader” events, and the evolution of viral variants (4–8).

**Figure 1:**
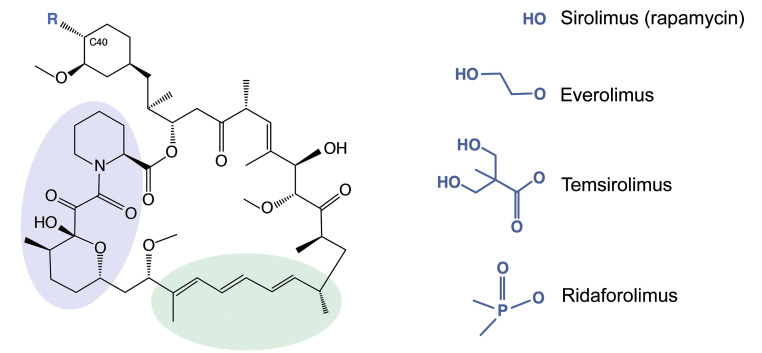
Rapamycin and its analogs share a macrolide structure but differ by the functional group present at carbon 40. Violet and green bubbles indicate the FKBP- and mTOR-binding sites, respectively. C40, carbon 40.

One group of compounds being considered for the treatment of COVID-19–related immunopathology are rapamycin (sirolimus, Rapamune) and rapamycin analogs (rapalogs) (9–20). As FDA-approved inhibitors of mTOR kinase, these macrolide compounds are used therapeutically to inhibit the processes of cancer, autoimmunity, graft-versus-host disease, atherosclerosis, and aging (21). Rapalogs, including everolimus (RAD-001), temsirolimus (Torisel, CCI-779), and ridaforolimus (deforolimus, AP-23573), were developed to decrease the half-life of rapamycin in vivo in order to minimize the systemic immunosuppression caused by rapamycin use, which is associated with increased susceptibility to infections (22–26). Differing by only a single functional group at carbon-40 (Figure 1), it is believed that rapamycin and rapalogs share the same molecular mechanism of action to inhibit mTOR kinase: they bind to FK506-binding proteins (FKBPs), and the resulting complex physically interacts with mTOR and disrupts its signaling (25, 27).

Activation of mTOR promotes cell growth, cell proliferation, and cell survival (28). In addition, mTOR activation promotes proinflammatory T cell differentiation, and mTOR inhibitors have been used to block lymphocyte proliferation and cytokine storm (29). Since respiratory virus infections like SARS-CoV-2 can cause disease by provoking hyperinflammatory immune responses that result in immunopathology (30–32), rapalogs are being tested as treatments to decrease viral disease burden. At least 3 active clinical trials have been designed to test the impact of rapamycin on COVID-19 severity in infected patients (NCT04461340, NCT04341675, and NCT04371640).

In addition to their potential utility for mitigating disease in individuals already infected by SARS-CoV-2, there are also calls to use rapalogs as antiviral agents to inhibit viral infection itself (i.e., as a prophylactic) (33). It was recently shown that rapalogs inhibit SARS-CoV-2 replication when added to cells after infection (34), attesting to a potential use of rapalogs as antiviral agents in infected individuals. Nonetheless, rapalogs are known to induce an immunosuppressed state in humans that is characterized by an increased rate of infections, including those caused by respiratory viruses. Furthermore, administration of rapamycin concurrently with virus challenge has been shown to promote influenza A replication in mice and to exacerbate viral disease (35, 36), but the mechanism was unknown. We previously found that exposure of human and murine cells to rapamycin induced the lysosomal degradation of a select group of cellular proteins, including the IFN-induced transmembrane (IFITM) proteins, and rendered cells more permissive to infection by influenza A virus and gene-delivering lentiviral vectors (37, 38). IFITM1, IFITM2, and IFITM3 are expressed constitutively in a variety of tissues, are further upregulated by type I, type II, and type III IFNs, and are important components of cell-intrinsic immunity, the antiviral network that defends individual cells against virus invasion (39, 40). Nonetheless, it remained to be determined how rapamycin-mediated regulation of intrinsic immunity affects host susceptibility to virus infection in vivo.

In this report, we show that rapalogs differentially counteract the constitutive and IFN-induced antiviral state in lung cells and increase permissiveness to SARS-CoV-2 infection. We found that the enhancing effect of rapalogs on SARS-CoV-2 infection was functionally linked to their capacity to trigger the degradation of IFITM proteins, particularly IFITM2 and IFITM3. By identifying a rapalog that lacks this activity, we found that IFITM protein turnover and SARS-CoV-2 infection enhancement were triggered by activation of TFEB, a master regulator of lysosome function that is regulated by mTOR. Administration of rapamycin to naive rodents prior to and after experimental SARS-CoV-2 infection increased virus replication and viral disease severity, indicating for the first time to our knowledge that suppression of intrinsic immunity by rapamycin contributes to its immunosuppressive properties in vivo.

## Results

### Select rapalogs promote SARS-CoV-2 infection and downmodulate IFITM proteins in lung cells.

To assess how rapamycin and rapalogs affect SARS-CoV-2 infection, we took advantage of a pseudovirus system based on HIV. This pseudovirus (HIV-CoV-2) is limited to a single round of infection, cell entry is mediated by SARS-CoV-2 spike, and infection of target cells is measured by luciferase activity. SARS-CoV-2 can enter cells via multiple routes, and sequential proteolytic processing of spike is essential to this process. SARS-CoV-2 spike is cleaved at a polybasic motif (RRAR) located at the S1/S2 boundary by furin-like proteases in virus-producing cells prior to release. Subsequently, the S2′ site is cleaved by the trypsin-like protease TMPRSS2 on the target cell surface or by cathepsins B and L in target cell endosomes, triggering membrane fusion at those sites (41–43).

**Figure 2:**
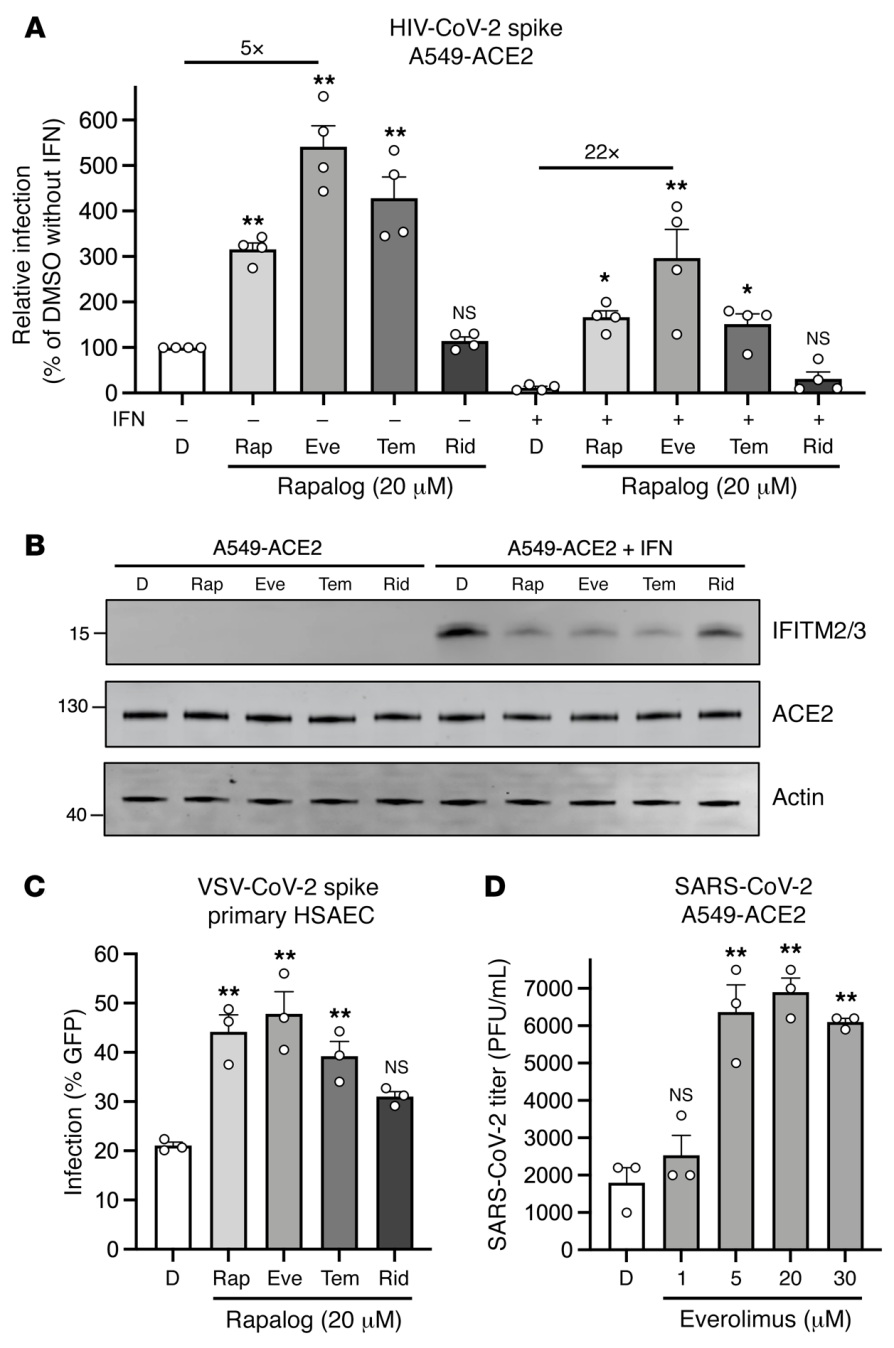
Rapalogs promote SARS-CoV-2 infection in lung epithelial cells to different extents by counteracting the intrinsic antiviral state. (**A**) A549-ACE2 cells were treated with or without type I IFN (250 U/mL) for 18 hours and then with 20 μM rapamycin (Rap), everolimus (Eve), temsirolimus (Tem), ridaforolimus (Rid), or an equivalent volume of DMSO (D) for 4 hours. HIV-CoV-2 (100 ng p24 equivalent) was added to cells, and infection was measured by luciferase activity 48 hours after infection. Luciferase units were normalized to 100 in the DMSO condition in the absence of IFN. (**B**) A549-ACE2 cells from **A** were subjected to SDS-PAGE and Western blot analysis. Immunoblotting was performed with anti–IFITM2/-3, anti-ACE2, and anti-actin (in that order) on the same nitrocellulose membrane. The numbers and tick marks indicate the size (kDa) and position of protein standards in ladders. (**C**) Primary hSAECs were treated with 20 μM rapamycin, everolimus, temsirolimus, ridaforolimus, or an equivalent volume of DMSO for 4 hours. VSV-CoV-2 (50 μL) was added to cells, and infection was measured by GFP expression 24 hours after infection using flow cytometry. (**D**) A549-ACE2 cells were treated with varying concentrations of everolimus or DMSO (equivalent to 30 μM everolimus) for 4 hours. SARS-CoV-2 (nCoV-WA1-2020; MN985325.1) was added to the cells at an MOI of 0.1, and infectious titers were measured in VeroE6 cells by calculating the TCID_50_ per milliliter of supernatants recovered 24 hours after infection. TCID_50_ (PFU/mL) values are shown. Means and the standard error were calculated from 3–4 experiments. *P < 0.05 and **P < 0.01, by 1-way ANOVA versus DMSO.

We previously found that a 4-hour pretreatment of cells with 20 μM quantities of rapamycin triggered the degradation of human IFITM3 and enhanced cellular susceptibility to influenza A virus infection (38). Therefore, we pretreated A549-ACE2 (transformed human lung epithelial cells that overexpress the SARS-CoV-2 receptor human ACE2) with 20 μM rapamycin, everolimus, temsirolimus, ridaforolimus, or DMSO (vehicle control) for 4 hours and then challenged cells with HIV-CoV-2. Interestingly, we found that rapalogs promoted spike-mediated infection to different extents: rapamycin, everolimus, and temsirolimus significantly enhanced infection (up to 5-fold), while ridaforolimus did not (Figure 2A). To determine whether rapalogs promote cell permissiveness to infection by upregulating dependency factors or by downregulating restriction factors, we performed the same experiment in cells pretreated with type I IFN. Whereas type I IFN suppressed infection by approximately 90%, the addition of rapamycin, everolimus, or temsirolimus resulted in the rescue of infection by up to 20-fold (Figure 2A). As a result, infection levels were partially restored to those achieved in the absence of IFN, with everolimus having the greatest boosting effect and ridaforolimus the least (Figure 2C). These results indicate that rapalogs differentially promoted SARS-CoV-2 spike-mediated infection by counteracting intrinsic antiviral defenses in lung cells to varying extents.

Type I IFN treatment of A549-ACE2 cells resulted in the upregulation of IFITM2 and IFITM3, as detected by an antibody recognizing both proteins in whole-cell lysates (Figure 2B). A549-ACE2 cells express low but detectable levels of IFITM2/-3 in the absence of IFN treatment (Supplemental Figure 1A; supplemental material available online with this article; https://doi.org/10.1172/JCI160766DS1). Consistent with our previous study, the addition of rapamycin resulted in a substantial reduction in IFITM2/-3 protein levels in cells. In a manner that mirrored the differential effects of rapalogs on pseudovirus infection, everolimus and temsirolimus greatly diminished IFITM2/-3 levels, whereas ridaforolimus reduced IFITM2/-3 levels to a lesser extent (Figure 2B and Supplemental Figure 1A). In contrast, ACE2 levels were not affected by IFN or by rapalog treatment. Therefore, rapamycin derivatives may facilitate infection by antagonizing constituents of intrinsic immunity, including IFITM2/-3, and this activity is determined by the chemical moiety found at carbon 40 of the macrolide structure.

To extend our findings to primary lung cells, we performed similar experiments in human small airway epithelial cells (hSAECs). Although these cells were not permissive to HIV-CoV-2, they were susceptible to infection by a pseudovirus based on vesicular stomatitis virus (VSV-CoV-2), whereby infection is reported by GFP expression. Pretreatment of hSAECs with rapalogs enhanced VSV-CoV-2 infection to varying extents, but as observed in A549-ACE2 cells, everolimus had the greatest effect and ridaforolimus the least. Endogenous IFITM3 was readily detected in hSAECs under basal conditions (in the absence of IFN), and its levels were downmodulated differentially by rapalogs. However, IFITM1 was barely detected, and IFITM2 was not detected at all (Supplemental Figure 1B). siRNA-mediated knockdown of IFITM3 in hSAECs resulted in enhanced VSV-CoV-2 infection, indicating that IFITM3 restricted spike-mediated infection in these cells (Supplemental Figure 1C). We also treated transformed nasal epithelial cells (UNCNN2TS) with rapalogs in order to assess the impact on endogenous IFITM3 levels. As observed in hSAECs, we found that downmodulation of IFITM3 occurred following treatment of UNCNN2TS cells with rapamycin, everolimus, or temsirolimus and, to a lesser extent, with ridaforolimus (Supplemental Figure 1D).

Since 20 μM doses of rapalogs promoted pseudovirus infection mediated by SARS-CoV-2 spike, we tested how pretreatment of A549-ACE2 cells with varying amounts of everolimus would affect infection by replication-competent SARS-CoV-2. We observed a dose-dependent enhancement (up to 4-fold) of the infectious SARS-CoV-2 yield in supernatants of infected cells (Figure 2D). Therefore, everolimus boosted pseudovirus infection and SARS-CoV-2 infection to similar extents. Since the spike protein is the only viral component shared between these 2 sources of infection, we conclude that rapalogs promoted infection by downmodulating intrinsic defenses acting at the infection stage of cellular entry.

### Rapalogs facilitate cell entry mediated by various viral fusion proteins.

**Figure 3:**
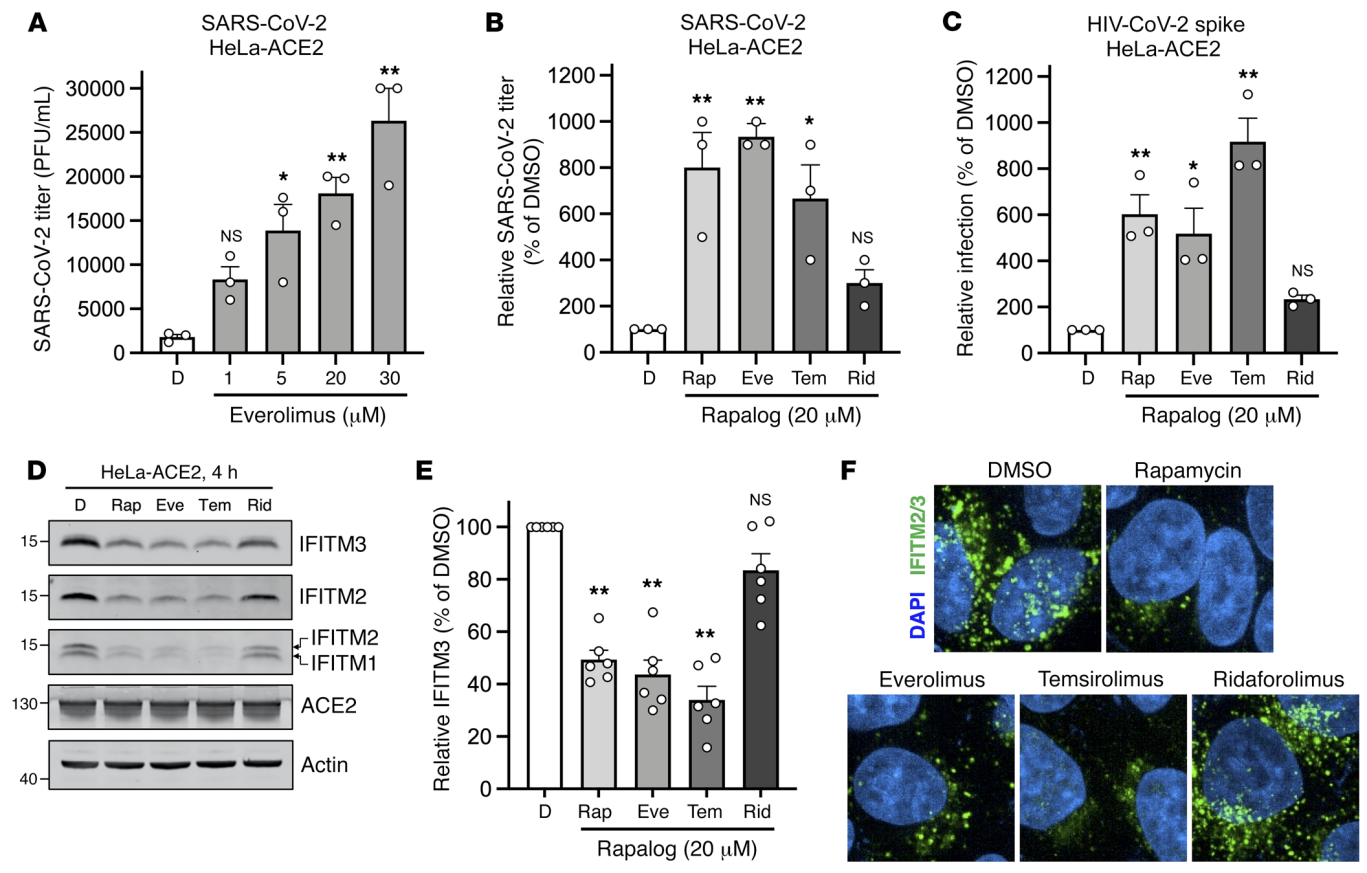
Rapalogs promote SARS-CoV-2 infection in HeLa-ACE2 cells. (**A**) HeLa-ACE2 cells were treated with varying concentrations of everolimus or DMSO for 4 hours. SARS-CoV-2 (nCoV-WA1-2020; MN985325.1) was added to cells at an MOI of 0.1, and infectious titers were measured in VeroE6 cells by calculating the TCID_50_ of supernatants recovered 24 hours after infection. TCID_50_ (PFU/mL) values are shown. (**B**) HeLa-ACE2 cells were treated with 20 μM rapamycin, everolimus, temsirolimus, ridaforolimus, or an equivalent volume of DMSO for 4 hours. SARS-CoV-2 (nCoV-WA1-2020; MN985325.1) was added to cells at an MOI of 0.1, and infectious titers were measured in VeroE6 cells by calculating the TCID_50_ per milliliter of supernatants recovered 24 hours after infection. TCID_50_ per milliliter values were normalized to 100 in the DMSO condition. (**C**) HeLa-ACE2 cells were treated with 20 μM rapamycin, everolimus, temsirolimus, ridaforolimus, or an equivalent volume of DMSO for 4 hours. HIV-CoV-2 (100 ng p24 equivalent) was added to cells, and infection was measured by luciferase activity 72 hours after infection. Luciferase units were normalized to 100 in the DMSO condition. (**D**) HeLa-ACE2 cells from **C** were subjected to SDS-PAGE and Western blot analysis. Immunoblotting was performed with anti-IFITM2, anti-IFITM1, anti-IFITM3, anti-ACE2, and anti-actin (in that order) on the same nitrocellulose membrane. (**E**) IFITM3 levels from **D** were normalized to actin levels and summarized from 5 independent experiments. (**F**) HeLa-ACE2 cells were treated with 20 μM rapamycin, everolimus, temsirolimus, ridaforolimus, or an equivalent volume of DMSO for 4 hours, and cells were fixed, stained with DAPI and anti–IFITM2/-3, and imaged by confocal immunofluorescence microscopy. Images represent stacks of 5 *Z*-slices, and 1 representative image is shown per condition. Original magnification, ×63. Means and the standard error were calculated from 3–6 experiments. *P < 0.05 and **P < 0.01, by 1-way ANOVA versus DMSO.

In order to gain a greater mechanistic understanding of the effects of rapalogs on SARS-CoV-2 infection, we took advantage of HeLa cells overexpressing ACE2 (HeLa-ACE2). We pretreated HeLa-ACE2 cells for 4 hours with increasing amounts of everolimus and then challenged them with SARS-CoV-2. Everolimus increased the titers of infectious virus released into supernatants in a dose-dependent manner, and to a greater extent than was observed for A549-ACE2 cells (Figure 3A). Furthermore, we found that pretreatment of cells with 20 μM doses of rapalogs enhanced SARS-CoV-2 titers to varying extents: rapamycin, everolimus, and temsirolimus significantly boosted SARS-CoV-2 infection (up to 10-fold), whereas ridaforolimus had less of an impact (Figure 3B). We also performed infections of HeLa-ACE2 with HIV-CoV-2 pseudovirus, and the results were similar: the impact of ridaforolimus was minimal, while the other 3 compounds significantly boosted spike-mediated infection (Figure 3C). To test the link between infection enhancement and downmodulation of IFITM proteins by rapalogs, we probed for levels of IFITM3, IFITM2, and IFITM1 by immunoblotting whole-cell lysates using specific antibodies. All IFITM proteins were readily detected in HeLa-ACE2 cells in the absence of IFN. IFITM3, IFITM2, and IFITM1 levels were downmodulated following treatment with rapamycin, everolimus, or temsirolimus (Figure 3D). The levels of IFITM3 were quantified over multiple experiments and are presented as an average. The results showed that all rapalogs induced significant decreases in IFITM3 protein, but ridaforolimus was least potent in this regard (Figure 3E). The loss of IFITM2/-3 protein was confirmed by confocal immunofluorescence microscopy of intact cells (Figure 3F). Furthermore, prolonged treatment (24 hours) of cells with everolimus or temsirolimus resulted in prolonged suppression of IFITM2 and IFITM3 protein levels (Supplemental Figure 2A). In contrast, ACE2 levels and ACE2 subcellular distribution were unaffected by rapalog treatment (Figure 3D and Supplemental Figure 2B). Furthermore, rapalogs did not significantly decrease cell viability under the conditions tested (Supplemental Figure 2C).

We previously showed that lysosomal degradation of IFITM3 triggered by rapamycin involves endosomal complexes required for transport (ESCRT) machinery and multivesicular body–lysosome (MVB-lysosome) fusion (38). We confirmed that depletion of IFITM proteins by rapalogs occurs at the posttranslational level and requires endolysosomal acidification, since bafilomycin A1 prevented their loss (Supplemental Figure 3, A and B). The process by which rapalogs trigger IFITM protein degradation resembles endolysosomal microautophagy, an autophagy pathway that does not require an autophagosome intermediate (44–46). Treatment of cells with U18666A, an inhibitor of MVB formation and microautophagy, mostly prevented IFITM3 turnover in the presence of rapalogs (Supplemental Figure 3B). In contrast, a selective inhibitor of vps34/PI3KC3 (essential for macroautophagy induction) did not prevent this turnover (Supplemental Figure 3, C and D). Therefore, rapamycin and select rapalogs reduced levels of IFITM proteins in cells by activating endosomal microautophagy.

**Figure 4:**
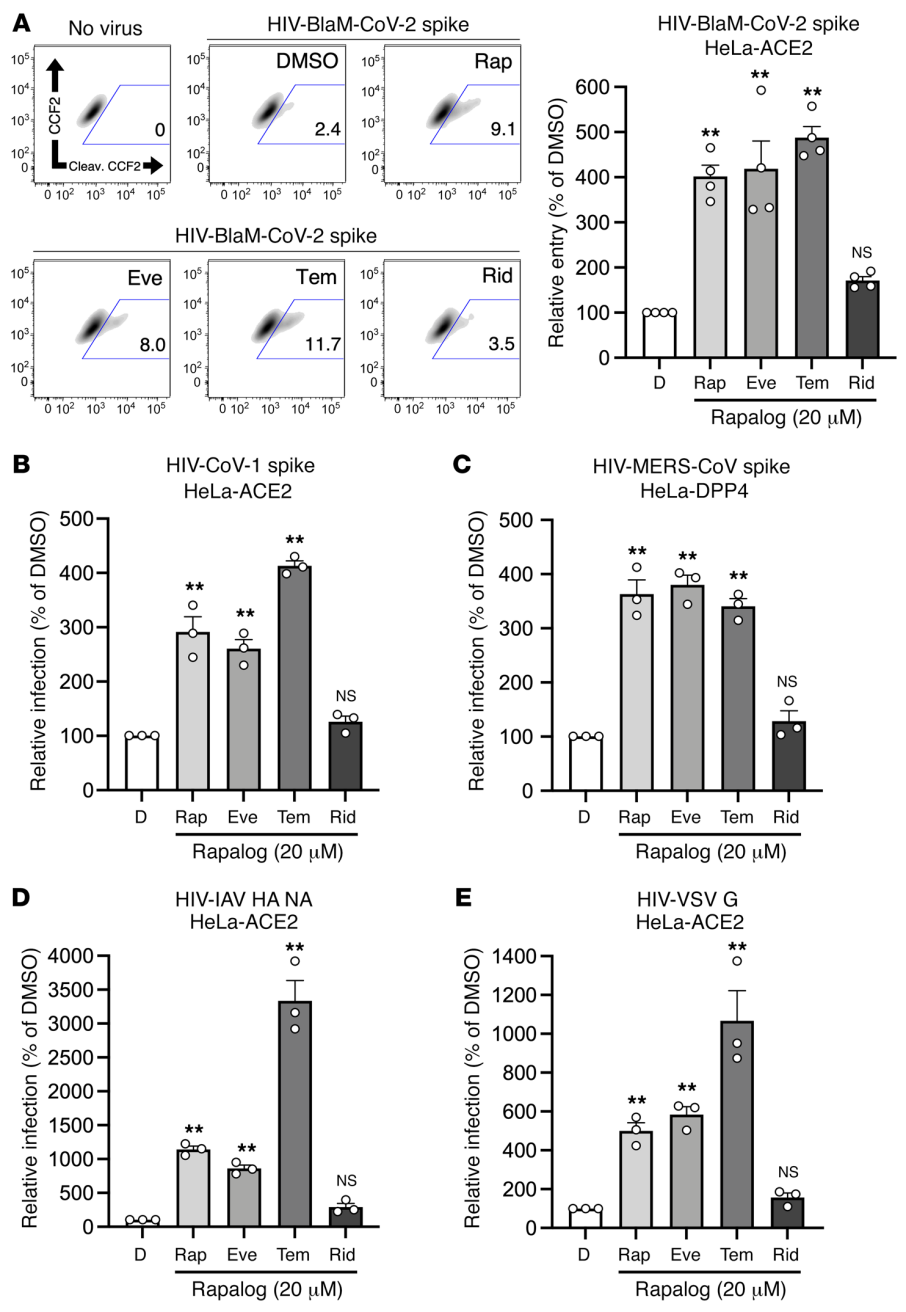
Rapalogs promote cell entry mediated by diverse viral fusion proteins. (**A**) HeLa-ACE2 cells were treated with 20 μM rapamycin, everolimus, temsirolimus, ridaforolimus, or an equivalent volume of DMSO for 4 hours. HIV-CoV-2 S pseudovirus incorporating BlaM-Vpr (HIV-BlaM-CoV-2) was added to cells for 2 hours and washed. Cells were incubated with CCF2-AM for an additional 2 hours and fixed. Cleaved CCF2 was measured by flow cytometry. Dot plots visualized as density plots from 1 representative experiment are shown on the left, and the percentage of CCF2^+^ cells that exhibited CCF2 cleavage is indicated. Summary data representing the average of 4 experiments are shown on the right. (**B**) HIV-CoV-1, (**C**) HIV-MERS-CoV, (**D**) HIV-IAV HA, or (**E**) HIV-VSV G was added to HeLa-ACE2 or HeLa-DPP4 cells as in **A**, and infection was measured by luciferase activity 72 hours after infection. Luciferase units were normalized to 100 in the DMSO condition. Means and the standard error were calculated from 3–4 experiments. **P < 0.01, by 1-way ANOVA versus DMSO.

Enveloped virus entry into cells is a concerted process involving virus attachment to the cell surface followed by fusion of cellular and viral membranes. Since IFITM proteins are known to inhibit virus-cell membrane fusion, we quantified the terminal stage of HIV-CoV-2 entry by tracking the cytosolic delivery of β-lactamase (BlaM) in single cells. We found that treatment of cells with rapamycin, everolimus, or temsirolimus resulted in enhanced HIV-CoV-2 entry, while treatment with ridaforolimus was less impactful (Figure 4A). To measure whether rapalogs promote the cell entry process driven by other coronavirus spike proteins, we produced HIV that incorporated spike from SARS-CoV (HIV-CoV-1) or MERS-CoV (HIV-MERS-CoV). Infections by both HIV-CoV-1 and HIV-MERS-CoV were elevated by rapalog treatment in HeLa-ACE2 and HeLa-DPP4 cells, respectively, although the extent of enhancement was lower than that observed with HIV-CoV-2 (Figure 4, B and C). Consistently, ridaforolimus was the least active among the rapalogs tested, and it did not significantly promote pseudovirus infection. Since we previously showed that rapamycin enhanced the cellular entry of influenza A virus and VSV glycoprotein (G) pseudotyped lentiviral vectors (38), we also assessed the infection of pseudoviruses incorporating hemagglutinin (HIV-HA) or VSV G (HIV–VSV G). Rapamycin, everolimus, and especially temsirolimus boosted HA- and VSV G–mediated infections (up to 30-fold and 11-fold, respectively) (Figure 4, D and E). Since IFITM proteins have been previously shown to inhibit infection by SARS-CoV, MERS-CoV, VSV, and influenza A virus (40), these data suggest that rapalogs promoted infection, at least in part, by lowering the barrier to virus entry imposed by IFITM proteins.

### IFITM2 and IFITM3 mediate the rapalog-sensitive barrier to SARS-CoV-2 infection in HeLa-ACE2.

**Figure 5:**
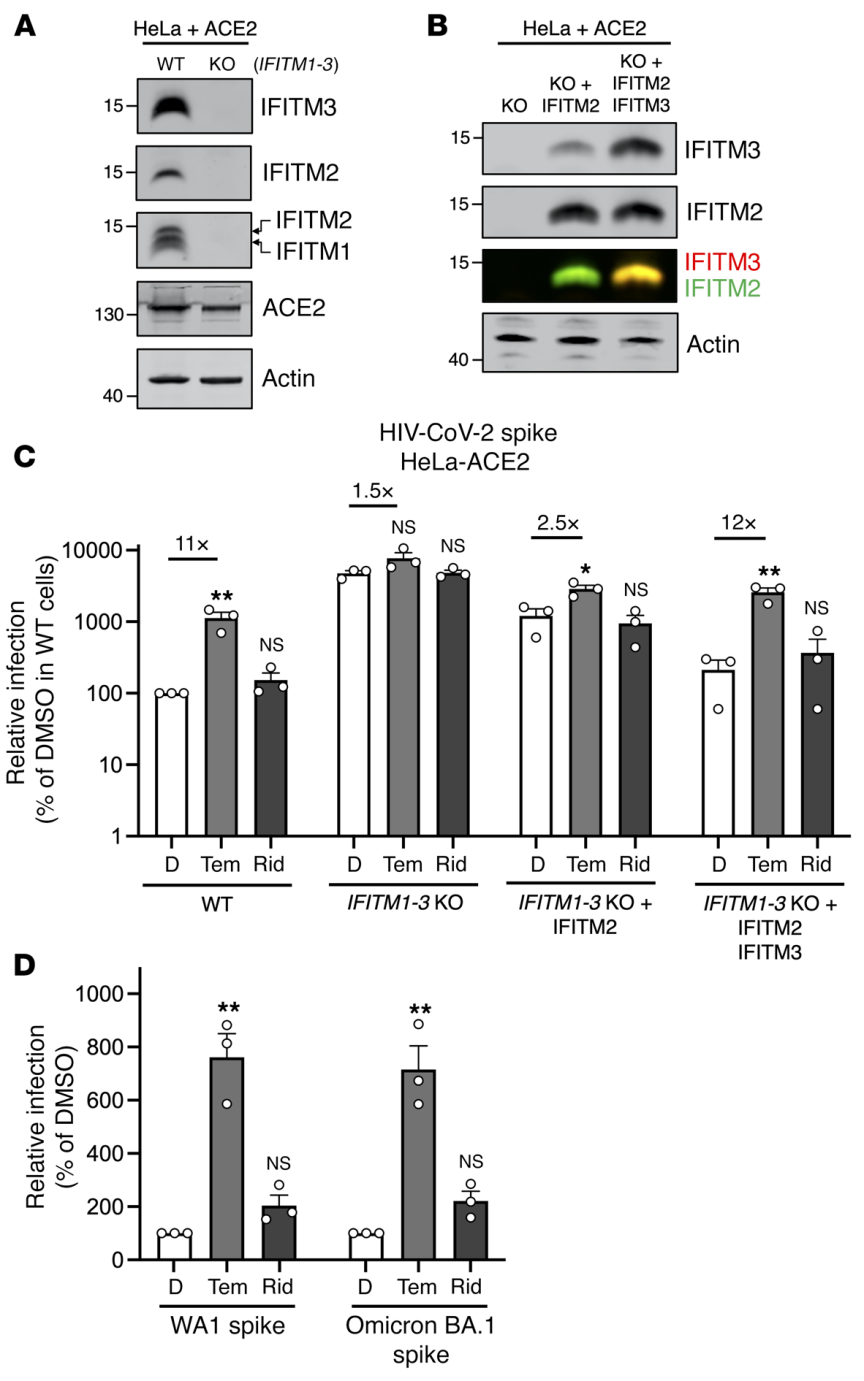
Select rapalogs enhance spike-mediated infection in HeLa-ACE2 cells by inhibiting IFITM2 and IFITM3. (**A**) WT HeLa and *IFITM1–3*–KO HeLa cells were transiently transfected with 0.150 μg pcDNA3.1-hACE2 for 24 hours. Whole-cell lysates were subjected to SDS-PAGE and Western blot analysis. Immunoblotting was performed with anti-IFITM2, anti-IFITM3, anti-IFITM1, anti-ACE2, and anti-actin (in that order) on the same nitrocellulose membrane. (**B**) HeLa *IFITM1–3*–KO cells were transfected with IFITM2 or IFITM2 plus IFITM3, and SDS-PAGE and Western blot analyses were performed. (**C**) HIV-CoV-2 was added to transfected cells from **B**, and infection was measured by luciferase activity 72 hours after infection. Luciferase units were normalized to 100 in WT HeLa cells treated with DMSO. (**D**) WT HeLa cells were transiently transfected with 0.150 μg pcDNA3.1-hACE2 for 24 hours. HIV-CoV-2 decorated with ancestral spike (WA1) or Omicron spike (BA.1) was added, and infection was measured by luciferase activity 48 hours after infection. Luciferase units were normalized to 100 in cells treated with DMSO for both pseudoviruses. Means and the standard error were calculated from 3 experiments. *P < 0.05 and **P < 0.01, by 1-way ANOVA versus the paired DMSO condition.

To formally test the link between rapalog-mediated depletion of IFITM proteins and entry by the SARS-CoV-2 spike protein, we used HeLa cells in which IFITM1, IFITM2, and IFITM3 were knocked out (*IFITM1–3–*KO) and introduced human ACE2 by transient transfection (Figure 5A). IFITM2 alone or IFITM2 and IFITM3 were restored in *IFITM1-3*–KO cells by transient overexpression (Figure 5B), and the cells were challenged with HIV-CoV-2. Relative to WT cells, HIV-CoV-2 infection was approximately 50-fold higher in *IFITM1-3*–KO cells, indicating that endogenous IFITM proteins restricted SARS-CoV-2 spike–mediated infection in this cell type. Furthermore, while temsirolimus significantly increased infection by 10-fold in WT cells, we observed little to no enhancement in *IFITM1-3*–KO cells (Figure 5C). Ectopic expression of IFITM2 inhibited infection and partially restored sensitivity to temsirolimus, while the combination of IFITM2 and IFITM3 restricted infection further and fully restored temsirolimus sensitivity. These findings indicate that temsirolimus promoted spike-mediated infection in HeLa-ACE2 cells by lowering the levels of endogenous IFITM2 and IFITM3. In accordance with the role played by endosomal IFITM2/-3 in protecting cells against SARS-CoV-2 infection (47), pseudovirus infection mediated by Omicron (BA.1) spike protein (which favors the endosomal route for entry) (48) was as sensitive to temsirolimus-mediated enhancement as infection mediated by ancestral (WA1) spike (Figure 5D). These results suggest that select rapalogs promoted SARS-CoV-2 infection by negating the antiviral action of IFITM2/-3 in endosomes.

Since human IFITM proteins have been reported to promote SARS-CoV-2 infection in certain cell types, including the lung epithelial cell line Calu-3 (49), we tested the effect of rapalogs on HIV-CoV-2 infection in this cell type. Here, in contrast to the enhancement observed in A549-ACE2 and HeLa-ACE2 cells, rapamycin, everolimus, and temsirolimus inhibited spike-mediated infection in Calu-3 cells, whereas ridaforolimus did not (Supplemental Figure 4A). Furthermore, rapamycin, everolimus, and temsirolimus reduced IFITM3 protein expression in this cell line, but ridaforolimus had a negligible effect (Supplemental Figure 4B). These results support the idea that the effect of rapalog treatment on spike-mediated infection is explained by their ability to induce the degradation of IFITM proteins, which inhibit SARS-CoV-2 infection in most contexts but enhance SARS-CoV-2 infection in Calu-3 cells for unknown reasons.

### Rapalogs differentially activate a lysosomal degradation pathway orchestrated by TFEB.

**Figure 6:**
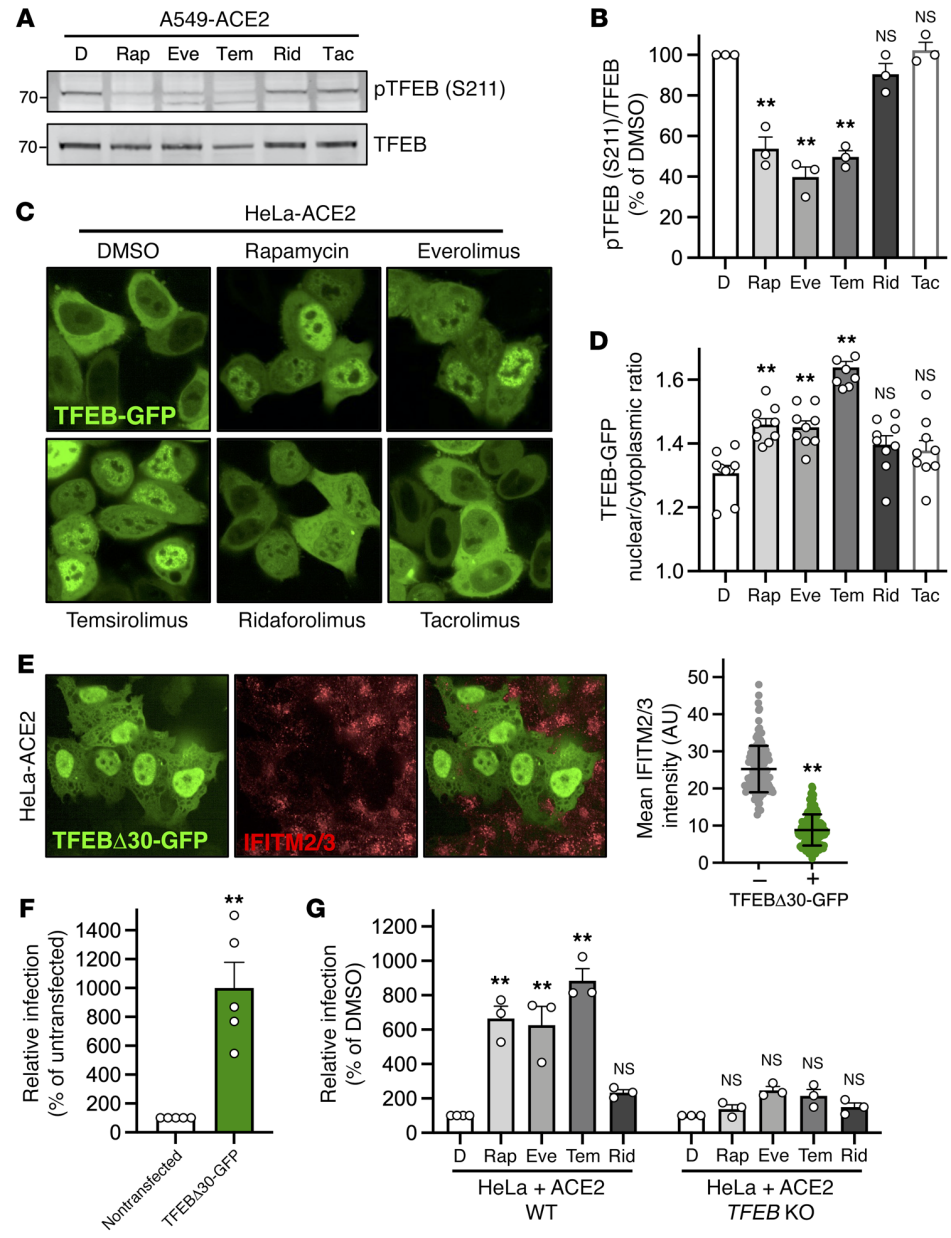
Nuclear TFEB triggers IFITM2/-3 turnover, promotes spike-mediated infection, and is required for enhancement of infection by rapalogs. (**A**) A549-ACE2 cells were treated with 20 μM rapamycin, everolimus, temsirolimus, ridaforolimus, tacrolimus (Tac), or DMSO for 4 hours, and whole-cell lysates were subjected to SDS-PAGE and Western blot analyses with anti-TFEB and anti–phosphorylated TFEB (anti-pTFEB) (S211). (**B**) pTFEB (S211) levels were divided by total TFEB levels and are summarized as an average of 3 experiments. (**C**) HeLa-ACE2 cells were transfected with TFEB-GFP for 24 hours, treated with rapamycin, everolimus, temsirolimus, ridaforolimus, or tacrolimus for 4 hours, stained with DAPI and CellMask (not shown), and imaged by high-content microscopy. Representative images are shown. Original magnification, ×40. (**D**) The ratio of nuclear to cytoplasmic TFEB-GFP was calculated in individual cells, and average ratios derived from 9 separate fields of view (each containing 20–40 cells) are shown. (**E**) HeLa-ACE2 cells were transfected with 0.5 μg TFEBΔ30-GFP for 24 hours, fixed, stained with anti–IFITM2/-3, and imaged by high-content microscopy (representative field on left). Original magnification, ×40. The average intensities of IFITM2/-3 levels in 150 GFP^–^ and 150 GFP^+^ cells were grouped from 2 transfections (right). (**F**) HeLa-ACE2 cells were transfected (or not) with 0.5 μg TFEBΔ30-GFP for 24 hours, and HIV-CoV-2 (100 ng p24 equivalent) was added. Infection was measured by luciferase 72 hours after infection. Luciferase units were normalized to 100 in the nontransfected condition. (**G**) WT HeLa or *TFEB*-KO HeLa cells were transfected with 0.3 μg pcDNA3.1-hACE2 for 24 hours and treated with 20 μM rapalogs/DMSO for 4 hours. HIV-CoV-2 (100 ng p24 equivalent) was added, and luciferase activity was measured 72 hours after infection. Luciferase units were normalized to 100 in the nontransfected condition. Means and the standard error were calculated from 3 (**A**), 5 (**F**), and 3 (**G**) experiments. **P < 0.01, by 1-way ANOVA (**B**, **D**, and **G**) and Student’s *t* test (**E** and **F**), versus DMSO or nontransfected conditions.

Since rapamycin and rapalogs are known to inhibit mTOR signaling by binding both mTOR and FKBP12 (and other FKBP members), we sought to determine whether mTOR binding and its inhibition are required for rapalog-mediated enhancement of SARS-CoV-2 infection. To that end, we tested the effect of tacrolimus (also known as FK506), a macrolide immunosuppressant that is chemically related to rapalogs but does not bind or inhibit mTOR. Instead, tacrolimus forms a ternary complex with FKBP12 and calcineurin to inhibit the signaling properties of the latter (50). In HeLa-ACE2 cells, a 4-hour treatment of 20 μM tacrolimus did not reduce IFITM2/-3 levels (Supplemental Figure 5A), nor did it boost HIV-CoV-2 infection (Supplemental Figure 5B). These results suggest that FKBP12 binding was not sufficient for drug-mediated enhancement of SARS-CoV-2 infection. These findings also suggest that the extent to which mTOR was inhibited may explain the different degrees to which infection was affected by the immunosuppressants examined in this study. Therefore, we surveyed the phosphorylation status of TFEB, a transcription factor that controls lysosome biogenesis and degradative processes carried out by lysosomes (51). mTOR phosphorylates TFEB at serine 211 (S211), which promotes its sequestration in the cell cytoplasm and decreases its translocation into the nucleus (51–53). Furthermore, this phosphorylation event was previously shown to be sensitive to inhibition by rapamycin and temsirolimus (52, 54). We found that rapamycin, everolimus, and temsirolimus significantly reduced S211 phosphorylation of endogenous TFEB in A549-ACE2 cells, while ridaforolimus did so to a lesser extent (Figure 6, A and B). Furthermore, we measured the subcellular distribution of TFEB-GFP in HeLa-ACE2 cells treated with different compounds and found that rapamycin, everolimus, and temsirolimus induced a significantly greater accumulation of TFEB-GFP in the nucleus (Figure 6, C and D). Therefore, nuclear translocation of TFEB was associated with IFITM2/-3 degradation and increased cellular susceptibility to SARS-CoV-2 spike–mediated infection.

We confirmed that 20 μM ridaforolimus did not inhibit S211 phosphorylation of TFEB in HeLa-ACE2 cells, whereas the same concentration of temsirolimus did (Supplemental Figure 6, A and B). To better understand why ridaforolimus displayed less activity with regard to enhancement of SARS-CoV-2 infection and inhibition of TFEB phosphorylation, we treated cells with increasing concentrations of ridaforolimus. Interestingly, we found that 30 μM ridaforolimus boosted infection to an extent similar to that seen with 20 μM temsirolimus, and 50 μM ridaforolimus boosted infection even further (Supplemental Figure 6C). Further cementing the link between infection enhancement and nuclear translocation of TFEB, we found that elevated concentrations of ridaforolimus, which resulted in increased infection, were also sufficient to inhibit TFEB phosphorylation (Supplemental Figure 6D). These findings indicate that, compared with other rapalogs, ridaforolimus is a less potent inhibitor of mTOR-mediated phosphorylation of TFEB, which may have important implications for the clinical use of ridaforolimus as an mTOR inhibitor in humans.

Consistent with a direct relationship between TFEB activation, IFITM2/-3 turnover, and spike-mediated cell entry, we found that ectopic expression of a constitutively active form of TFEB lacking the first 30 amino-terminal residues (51) was sufficient to both trigger IFITM2/-3 loss from cells (Figure 6E) and increase susceptibility to HIV-CoV-2 infection (Figure 6F). By combining transfection of the constitutively active form of TFEB with temsirolimus treatment, we found that IFITM2/-3 levels were strongly suppressed, irrespective of whether TFEB was detected. This confirms that TFEB and rapalogs were functionally redundant and operated in the same pathway to negatively regulate IFITM2/-3 levels (Supplemental Figure 7A). Finally, we took advantage of TFEB-deficient cells to formally address the role that TFEB activation plays during rapalog-mediated enhancement of infection (Supplemental Figure 7B). While rapamycin, everolimus, and temsirolimus significantly boosted HIV-CoV-2 infection in WT HeLa cells transfected with ACE2, no significant enhancement was observed in *TFEB*-KO HeLa cells (Figure 6G). In summary, our results using functionally divergent rapalogs revealed what we believe to be a previously unrecognized immunoregulatory role played by the mTOR/TFEB/lysosome axis that affects the cell entry of SARS-CoV-2 and other viruses.

### Rapamycin enhances SARS-CoV-2 replication in primary human nasal epithelia and promotes viral disease in animal models.

**Figure 7:**
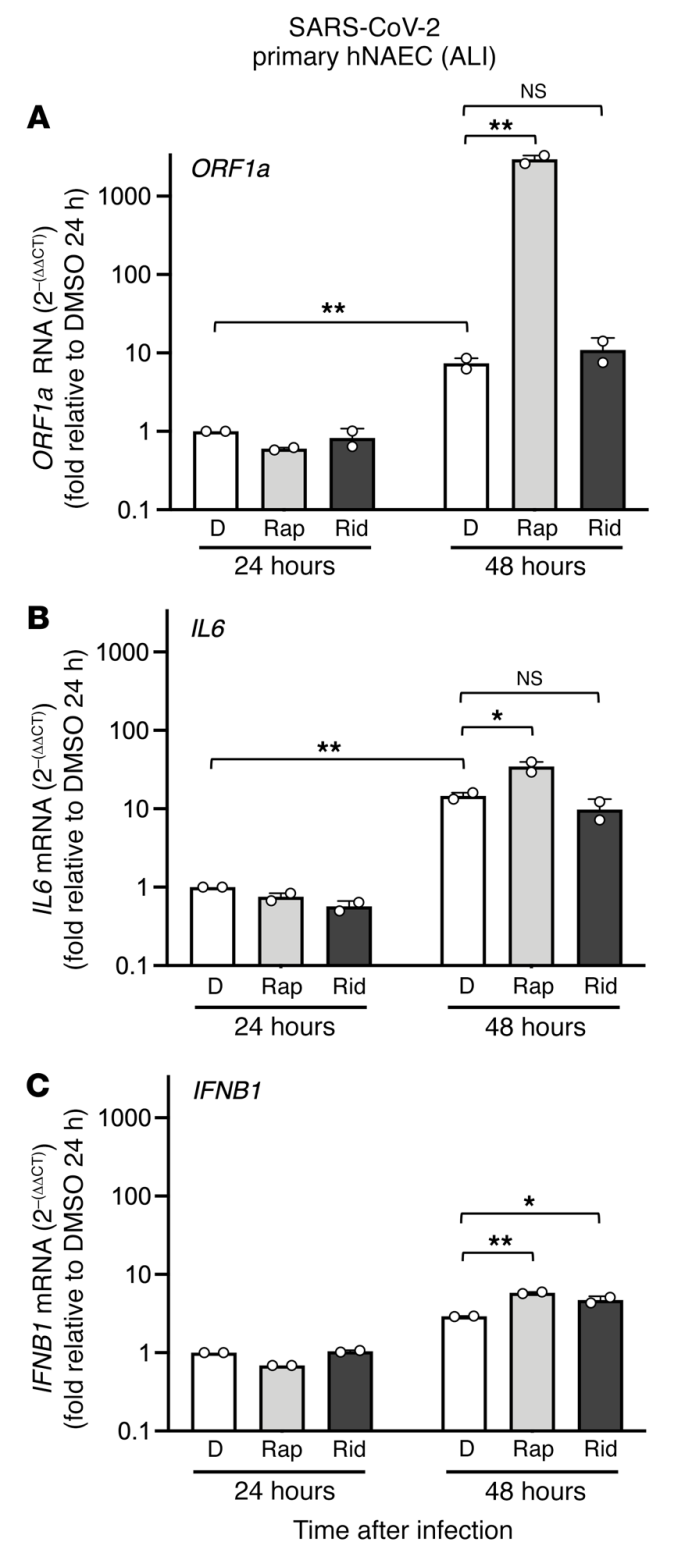
Rapamycin increases susceptibility of primary hNAECs to SARS-CoV-2 infection while limiting proinflammatory cytokine induction. Primary hNAECs pooled from 12 donors were cultured at the air-liquid interface (ALI) for 30–60 days and were infected with 5 × 10^5^ PFU SARS-CoV-2 (WA1). Twenty-four hours and 48 hours after infection, TRIzol was added to cells, and total RNA extraction was performed. RT-qPCR was performed using primers and probes specific to viral *ORF1A* (**A**), cellular *IL6* (**B**), and cellular *IFNB1* (**C**). Means and the standard error were calculated from 2 experiments (infection of pooled cells from 12 human donors was performed in duplicate). Relative RNA levels are presented as the comparative Ct method with *ACTB* serving as an endogenous control. RNA levels present in the DMSO condition at 24 hours were normalized to 1. *ORF1A* was not detected in noninfected cells. *P < 0.05 and **P < 0.01, by 1-way ANOVA. rel., relative.

Our findings from SARS-CoV-2 and pseudovirus infection of human cells demonstrate that rapamycin, everolimus, and temsirolimus could suppress intrinsic immunity at the posttranslational level, while ridaforolimus showed decreased potency in this regard. However, whether these compounds would be functionally divergent when administered in vivo was unclear. To closely approximate the conditions under which SARS-CoV-2 infects and replicates within the human respiratory tract, we tested how rapamycin or ridaforolimus affected SARS-CoV-2 replication in primary human nasal epithelial cells cultured at the air-liquid interface, a tissue model that recapitulates the 3D physiology of the upper airway. Measurement of viral *ORF1A* RNA by reverse transcription quantitative PCR (RT-qPCR) was used to assess the levels of viral transcripts 24 and 48 hours after infection, whereas *IL6* and *IFNB1* mRNA levels were measured to assess the concomitant induction of cytokines. The levels of *ORF1A* significantly increased between 24 and 48 hours after infection, suggesting that these cells support virus replication (Figure 7A). Furthermore, we found that rapamycin significantly enhanced virus replication (400-fold) 48 hours after infection, while ridaforolimus did not (Figure 7A). Consistent with enhanced virus replication in those cells, *IL6* and *IFNB1* transcripts were significantly elevated by rapamycin (Figure 7, B and C). However, since rapamycin elevated viral *ORF1A* by 400-fold but only increased cellular *IL6* and *IFNB1* by 2.5-fold or less, these results suggest that rapamycin increased cellular susceptibility to SARS-CoV-2 infection, while limiting inflammatory cytokine induction in response to infection.

**Figure 8:**
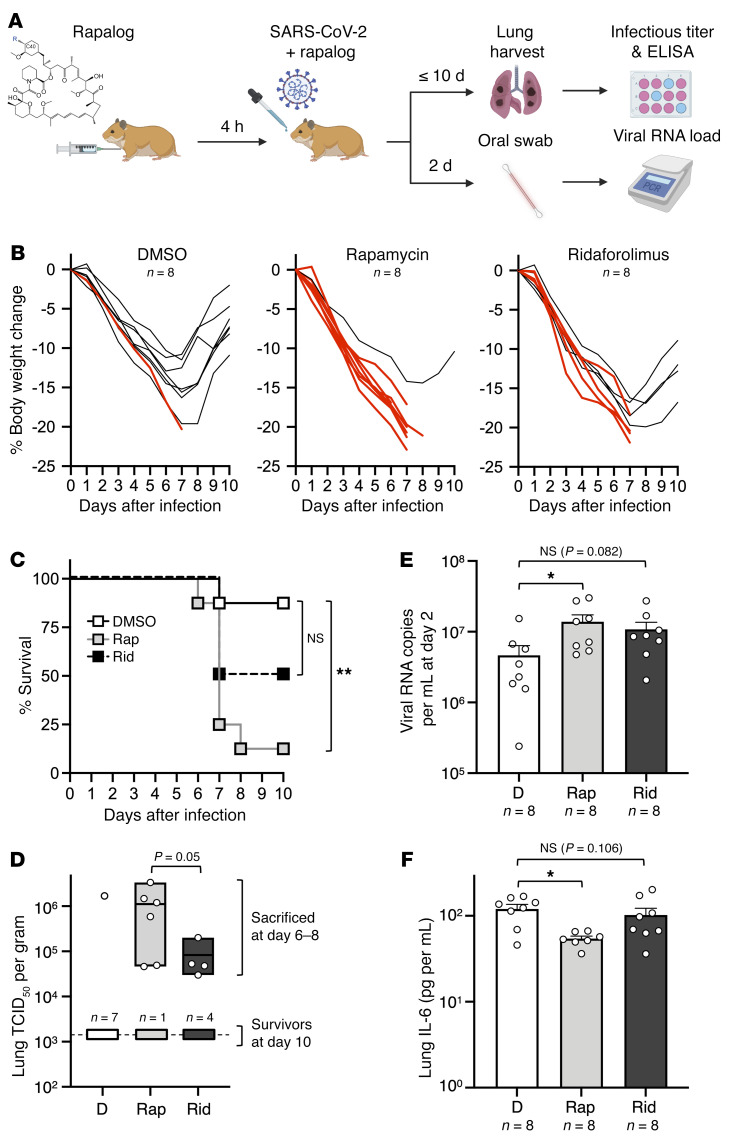
Rapamycin injection into hamsters intensifies viral disease during SARS-CoV-2 infection. (**A**) Golden Syrian hamsters were injected intraperitoneally with 3 mg/kg rapamycin, ridaforolimus, or an equivalent amount of DMSO (*n* = 4 animals per group). Four hours later, hamsters were infected intranasally with 6 × 10^3^ PFU SARS-CoV-2. On post-infection day 2, half of the animals received a second injection of rapamycin, ridaforolimus, or DMSO. Oral swabs were taken and used for measurement of oral viral RNA load by RT-qPCR. On post-infection day 10 (or earlier if more than 20% weight loss or agonal breathing was detected), hamsters were euthanized, and lungs were harvested for determination of infectious virus titers by TCID_50_ assay and IL-6 ELISA. (**B**) Individual body weight trajectories for each treatment group are plotted by day post-infection. Red lines indicate animals that required euthanasia at humane endpoints (more than 20% weight loss or agonal breathing). (**C**) Kaplan-Meier survival curves were generated according to the dates of euthanasia (or in 1 case, when the animal was found dead). (**D**) Infectious virus titers in lungs were determined by TCID_50_ in Vero-TMPRSS2 cells. Data are depicted as floating bars (minimum, maximum, and mean are shown). (**E**) Viral RNA copy numbers were determined by RT-qPCR from oral swab 2 days after infection. Data are depicted as box-and-whisker plots. (**F**) IL-6 protein levels in lungs were determined using a hamster IL-6 ELISA kit. Statistical analysis in **C** was performed by comparing survival curves between rapamycin and DMSO or ridaforolimus and DMSO treatments using the log-rank (Mantel-Cox) test. Statistical analysis in **D** was performed by comparing all individual animals (survivors and euthanized) in the rapamycin and ridaforolimus treatment groups using the Mann-Whitney *U* test. Statistical analysis in **E** and **F** was performed by 1-way ANOVA. *P < 0.05 and **P < 0.01. Illustration created with BioRender.com.

On the basis of these findings, we next tested how intraperitoneal injection of rapamycin or ridaforolimus would affect SARS-CoV-2 replication and disease course in naive hamsters (Figure 8A). Hamsters are a permissive model for SARS-CoV-2 because hamster ACE2 is sufficiently similar to human ACE2 to support productive infection. Furthermore, hamsters exhibit severe disease characterized by lung pathology when high viral loads are achieved (55). Eight hamsters were randomly allocated to each treatment group (rapamycin, ridaforolimus, or DMSO), and all received an intraperitoneal injection (3 mg/kg) 4 hours prior to intranasal inoculation with SARS-CoV-2 WA1. Furthermore, half of the hamsters in each group received a second injection on day 2 after infection. As an indicator of viral disease, we tracked weight loss for 10 days, or less if the hamster met the requirements for euthanasia (loss of 20% or more of its body weight or signs of respiratory distress such as agonal breathing). We observed that hamsters receiving 2 injections did not exhibit significantly different rates of weight loss compared with those receiving a single injection (Supplemental Figure 8A). As a result, we consolidated the hamsters into 3 groups of 8 according to whether they received rapamycin, ridaforolimus, or DMSO. In addition to monitoring weight and breathing over the course of infection, disease scores (referred to here as “COVID scores”) were generated daily for each hamster. Scoring reflected the extent of coat ruffling, hunched posture, lethargic state, and weight loss, and mean scores were compiled for each group. In agreement with the increased occurrence of morbidity necessitating euthanasia (Figure 8B), the disease scores were higher on average for rapamycin- and ridaforolimus-treated hamsters relative to those treated with DMSO (Supplemental Figure 8B and Supplemental Table 1). Between post-infection days 6 and 8, one (1 of 8) of the hamsters treated with DMSO exhibited severe morbidity necessitating euthanasia compared with 7 of 8 of the hamsters treated with rapamycin (Figure 8, B and C). Meanwhile, 4 of 8 of the hamsters treated with ridaforolimus met the requirements for euthanasia. Survivors in all 3 groups of hamsters recovered their weight after post-infection day 7, and we detected no infectious virus from the lungs of these hamsters on day 10 (Figure 8D). Overall, the hamsters treated with rapamycin had significantly reduced survival compared with the DMSO-treated hamsters, whereas survival of the ridaforolimus-treated animals was decreased but did not differ significantly (Figure 8C).

The lungs of hamsters euthanized because of morbidity had high infectious virus titers, suggesting that morbidity was caused by viral pathogenesis (the lungs of 1 hamster treated with rapamycin were not examined because it was found dead following infection) (Figure 8D). In general, hamsters treated with rapamycin exhibited significantly higher infectious virus titers in lungs than did those treated with ridaforolimus (Figure 8D). In addition, early SARS-CoV-2 replication was measured by RT-qPCR from oral swabs. We found that hamsters injected with rapamycin exhibited significantly higher viral RNA levels in the oral cavity on post-infection day 2 compared with animals injected with DMSO (Figure 8E). In contrast, viral RNA levels in hamsters injected with ridaforolimus were elevated relative to the DMSO group, but they did not differ significantly. Consistent with the known inhibitory effects of rapamycin on cytokine signaling (29), we detected significantly less IL-6 protein in the lungs of hamsters treated with rapamycin, whereas ridaforolimus did not cause a reduction in IL-6 (Figure 8F). Overall, these results demonstrate that rapamycin administration significantly increased host susceptibility to SARS-CoV-2 infection and virus-induced morbidity in a manner that was not associated with an enhanced proinflammatory state.

These conclusions were supported by our histopathological analysis of lungs, which indicated that lung damage occurred in all infected hamsters, especially those that needed to be humanely euthanized. All hamsters, regardless of treatment group, exhibited signs of lung hyperplasia and mixed or mononuclear inflammation, whereas some hamsters were found to have lung edema, hypertrophy, fibrosis, or syncytial cell formation. Hamsters requiring euthanasia, regardless of treatment group, showed the additional signs of moderate-to-severe lung hemorrhage, while minor hemorrhaging was apparent in only 2 hamsters that survived until day 10 after infection (Supplemental Table 1 and Supplemental Data). On average, rapamycin treatment increased the severity of lung edema, hemorrhage, hypertrophy, and inflammation in the infected hamsters (Supplemental Figure 8C). Furthermore, since we detected the highest viral loads in the lungs of morbid hamsters (Figure 8D), lung dysfunction (acute respiratory distress syndrome) caused by virus replication was the likely cause of morbidity in these hamsters. This is further supported by instances of agonal breathing in some of the infected hamsters, which necessitated euthanasia (Supplemental Table 1).

**Figure 9:**
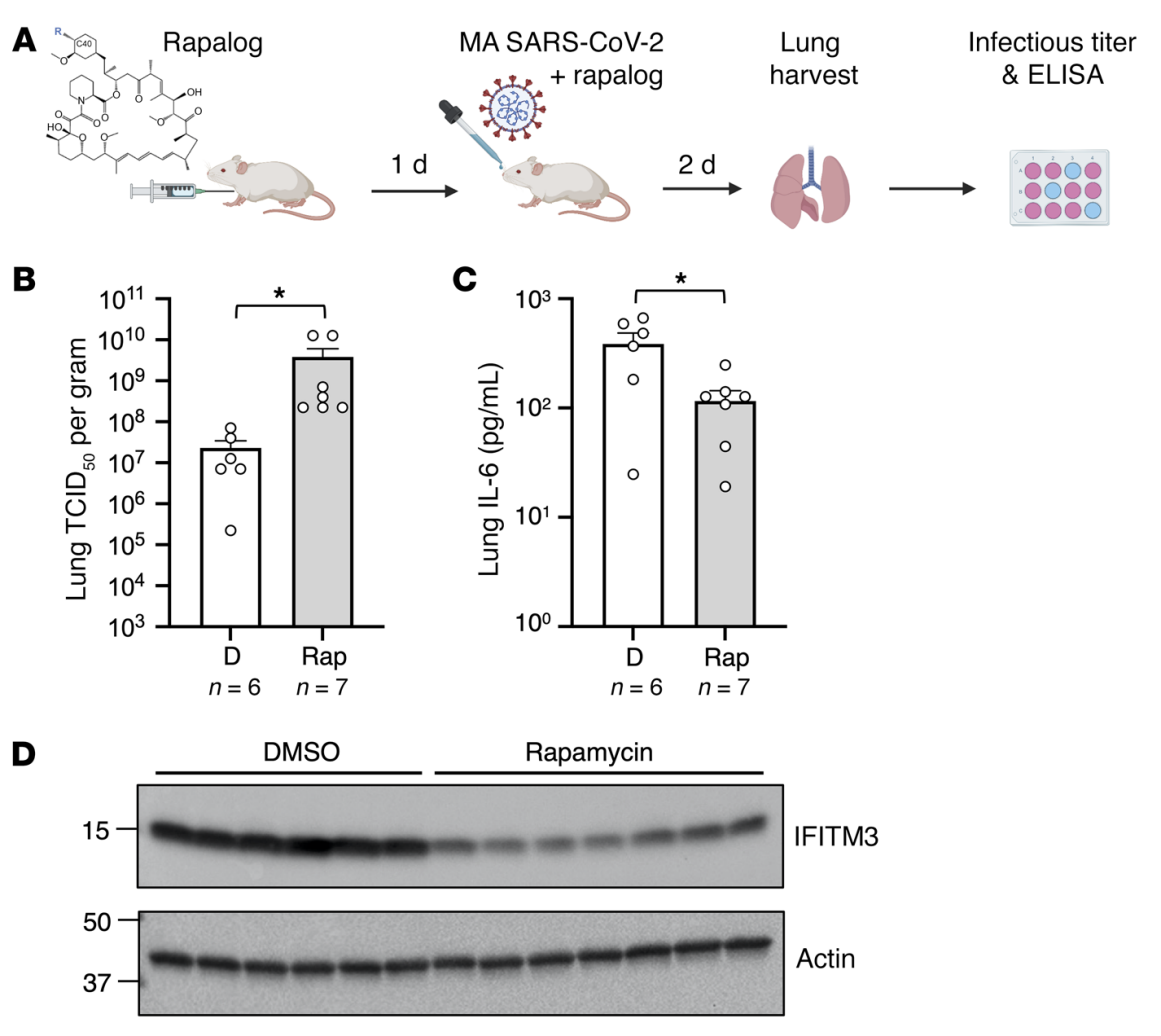
Rapamycin injection into mice downmodulates IFITM3 in lungs and boosts MA SARS-CoV-2 titers. (**A**) C57BL/6 mice were injected with 3 mg/kg rapamycin or an equivalent amount of DMSO (*n* = 6 and *n* = 7 mice per group, respectively). The following day, mice were infected intranasally with 6 × 10^4^ TCID_50_ MA SARS-CoV-2. Mice received second and third injections of rapamycin or DMSO on the day of infection and on day 1 after infection. (**B**) Lungs were harvested from infected mice upon euthanasia on day 2 after infection. Infectious viral loads were determined by TCID_50_ (**B**), and IL-6 protein was measured using a mouse IL-6 ELISA kit (**C**). The geometric mean TCID_50_ per gram was calculated for each treatment group. *P < 0.05, by Mann-Whitney *U* test versus DMSO. (**D**) Lung homogenates (3 μg) from mice injected with rapamycin or DMSO were subjected to SDS-PAGE and Western blot analyses. Immunoblotting was performed with anti-fragilis/anti-IFITM3 (ab15592) and anti-actin.

Rapamycin was previously shown to promote morbidity from influenza A infection in mice (36, 56). Moreover, we previously found that murine IFITM3 is sensitive to depletion by rapamycin (38). To determine whether rapamycin promotes host susceptibility to SARS-CoV-2 infection in mice, we injected C57BL/6 mice with rapamycin or DMSO prior to and after challenge with mouse-adapted (MA) SARS-CoV-2 (Figure 9A). In this model, we did not observe significant weight loss for up to 5 days following infection (Supplemental Figure 9). Lungs from mice in both groups were harvested uniformly on post-infection day 2, and we found that virus titers were significantly increased (144-fold) in rapamycin-treated mice compared with DMSO-treated mice (Figure 9B). As observed in hamsters, IL-6 levels were significantly reduced in lungs from rapamycin-treated mice, despite enhanced virus titers (Figure 9C). Furthermore, murine IFITM3 protein levels were reduced in the lungs of mice injected with rapamycin compared with the levels found in DMSO-treated mice (Figure 9D). Together, these findings support the conclusion that rapamycin downmodulated cell-intrinsic barriers to SARS-CoV-2 infection in vivo and, as a result, enhanced virus replication and viral disease.

## Discussion

By assessing their impact on infection at the single-cell and whole-organism level, we draw attention to an immunosuppressive property of rapamycin and some rapalogs that acts on cell-intrinsic immunity and increases cellular susceptibility to infection by SARS-CoV-2 and likely other pathogenic viruses. The side effects of rapalogs used in humans, including increased risk of respiratory tract infections, are regularly attributed to immunosuppression of adaptive immunity (57). Indeed, rapalogs have been used to mitigate systemic immunopathology caused by T cell responses, and this is one reason why they are being tested for therapeutic benefit in patients with COVID-19. However, since rapamycin was injected into immunologically naive hosts prior to and soon after virus challenge and followed for no more than 10 days, it is unlikely that rapalogs modulated adaptive immunity against SARS-CoV-2 in our experiments. While immunomodulation of adaptive immunity by rapalogs may provide benefit for patients who already have COVID-19, preexisting rapalog use may enhance host susceptibility to infection and disease by counteracting cell-intrinsic immunity.

The injection dose of rapamycin or ridaforolimus (3 mg/kg) that we administered to hamsters and mice, when adjusted for body surface area and an average human weight of 60 kg (58), equates to a dose of approximately 15 mg for humans. This dose is similar to those administered to humans in clinical settings, such as the use of rapamycin for the treatment of glioblastoma (up to 10 mg daily for multiple days), the use of temsirolimus for the treatment of renal cell carcinoma (25 mg once weekly), or the use of everolimus for the treatment of tuberous sclerosis (TS), a genetic disorder resulting in hyperactivation of mTOR (10 mg daily, continuously) (23, 59–61). Interestingly, a case report detailed the deaths of 2 patients with TS (a father and daughter), who, despite discontinuing everolimus upon detection of SARS-CoV-2 infection, died from severe COVID-19 in late 2020 (61). Our findings detailing the suppression of cell-intrinsic immunity by rapalogs raise the possibility that their use may predispose individuals to SARS-CoV-2 infection and severe forms of COVID-19. More generally, our findings provide insight into how rapamycin and rapalogs may lead to unintended immunocompromised states and increase human susceptibility to multiple viral infections.

**Figure 10:**
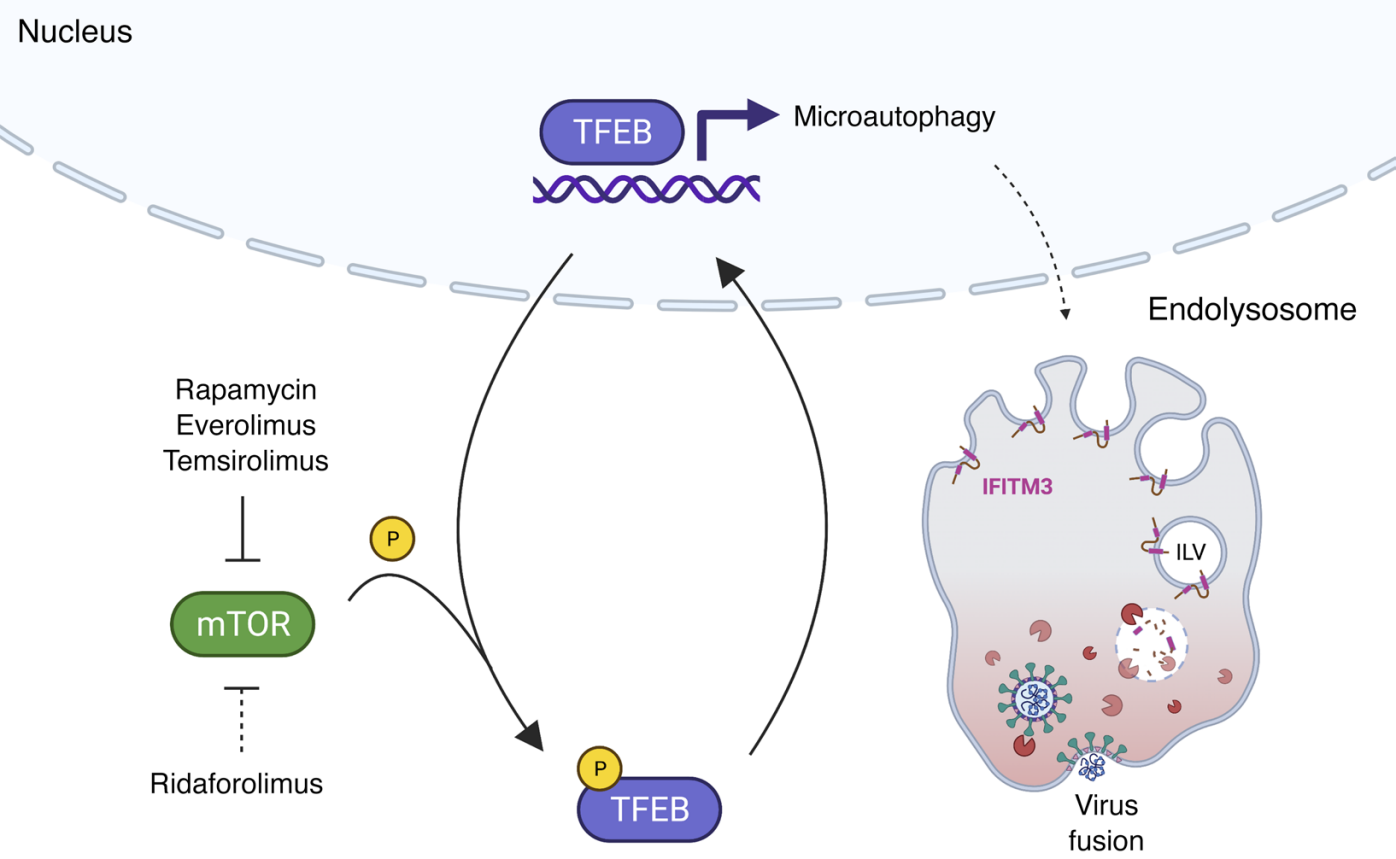
Model for rapalog-mediated enhancement of SARS-CoV-2 infection. Rapamycin, everolimus, and temsirolimus potently inhibit the phosphorylation of TFEB by mTOR, while ridaforolimus is a less potent inhibitor. As a result, TFEB translocates into the nucleus and induces the expression of genes involved in lysosomal functions, including genes involved in autophagy-related pathways. Nuclear TFEB triggers a microautophagy pathway that results in accelerated degradation of the membrane proteins IFITM2 and IFITM3. Loss of IFITM2/-3 promotes SARS-CoV-2 entry into cells by facilitating fusion between viral membranes and cellular membranes. The illustration was created with BioRender.com.

By leveraging the differential functional properties of rapalogs, we reveal how the mTOR/TFEB/lysosome axis affected intrinsic resistance to SARS-CoV-2 infection. Specifically, rapamycin and select rapalogs (everolimus and temsirolimus) promoted infection at the cell entry stage, and this was functionally linked to nuclear accumulation of TFEB and the lysosomal degradation of IFITM proteins by endosomal microautophagy (Figure 10). While mTOR phosphorylates TFEB at S211 to promote the sequestration of TFEB in the cytoplasm, the phosphatase calcineurin dephosphorylates TFEB at this position to promote nuclear translocation (62). Therefore, the extent to which different rapalogs promote nuclear TFEB accumulation may be a consequence of differential mTOR inhibition and/or differential calcineurin activation. Calcineurin is activated by calcium release through the lysosomal calcium channel TRPML1 (also known as mucolipin 1) (62), and, interestingly, it was shown that rapamycin and temsirolimus, but not ridaforolimus, promote calcium release by TRPML1 (54). Therefore, it is worth examining whether TRPML1 or related lysosomal calcium channels are required for the effects of rapalogs on virus infection. Overall, our findings reveal what to our knowledge is a previously unrecognized mechanism by which TFEB promotes virus infections via inhibition of cell-intrinsic defenses restricting virus entry. We show that nuclear TFEB induced the degradation of IFITM proteins, but it may also trigger the loss or relocalization of other antiviral factors that remain to be uncovered. Furthermore, TFEB-mediated induction of dependency factors, such as cathepsin L, is a likely partial contributor to the overall impact of rapalogs on SARS-CoV-2 infection. Overall, this work identifies TFEB as a therapeutic target, and inhibitors that limit the levels of nuclear TFEB could be mobilized for broad-spectrum antiviral activity.

We previously demonstrated that the treatment of cells with micromolar quantities of rapamycin induces the lysosomal degradation of IFITM2/-3 via a pathway that is independent of macroautophagy yet dependent on ESCRT-mediated sorting of IFITM2/-3 into intraluminal vesicles of late endosomes/MVB (38). This MVB-mediated degradation pathway is also referred to as microautophagy, which occurs directly on endosomal or lysosomal membranes and involves membrane invagination (63). In both yeast and mammalian cells, microautophagy is characterized by ESCRT-dependent sorting of endolysosomal membrane proteins into intraluminal vesicles followed by their degradation by lysosomal hydrolases (64). While microautophagy selectively targets ubiquitinated endolysosomal membrane proteins, cytosolic proteins can also be nonselectively internalized into intraluminal vesicles and degraded (65, 66). Interestingly, microautophagy is known to be regulated by mTOR (67, 68), and mTOR inhibition triggers a ubiquitin- and ESCRT-dependent turnover of vacuolar (lysosomal) membrane proteins in yeast (69, 70). Overall, our findings suggest that select rapalogs induce a rapid, TFEB-dependent, endolysosomal membrane remodeling program known as microautophagy, and IFITM proteins are among the client proteins subjected to this pathway. The full cast of cellular factors that orchestrate this selective degradation program in mammalian cells and the other client proteins subjected to it will need to be uncovered. Interestingly, the E3 ubiquitin ligase NEDD4 was previously shown to ubiquitinate IFITM2 and IFITM3 and to induce their lysosomal degradation in mammalian cells (71, 72), whereas Rsp5, the yeast ortholog of NEDD4, was shown to ubiquitinate vacuolar proteins turned over by microautophagy in yeast (73). Therefore, rapamycin and select rapalogs may upregulate NEDD4 function, resulting in selective degradation of a subset of the cellular proteome that includes IFITM proteins. Indeed, NEDD4 and the related NEDD4L are among the known target genes regulated by TFEB (74).

The relationship between IFITM proteins and human coronaviruses is complex. It was previously shown that IFITM3 facilitates replication of the seasonal coronavirus hCoV-OC43 (75), and we and others recently showed that SARS-CoV-1 and SARS-CoV-2 infection is inhibited by ectopic and endogenous IFITM1, IFITM2, and IFITM3 from mice and humans (47, 76–79). Intriguingly, mutants of human IFITM3 that lack the capacity to internalize into endosomes lost antiviral activity and promoted SARS-CoV-2 and MERS-CoV infection, revealing that IFITM3 can either inhibit or enhance infection depending on its subcellular localization (47, 80). Furthermore, 1 study reported that endogenous human IFITM proteins promote infection by SARS-CoV-2 in certain human tissues, possibly by acting as interaction partners and docking platforms for the viral spike protein (49). Overall, the net effect of human IFITM proteins on SARS-CoV-2 infection in vivo remains unclear. However, the impact of rapamycin in our experimental SARS-CoV-2 infections of hamsters and mice suggests that rapamycin-mediated loss of IFITM proteins favors virus infection and viral disease, consistent with the idea that IFITM proteins perform antiviral roles against SARS-CoV-2 in those species. Accordingly, it was recently demonstrated that mouse IFITM3 protects mice from viral pathogenesis following MA SARS-CoV-2 infection (81).

Other lines of evidence support an antiviral role for IFITM proteins during SARS-CoV-2 infection in humans. While SARS-CoV-2 infection has been shown to cause deficiencies in IFN synthesis and IFN response pathways, administration of type I IFN in vivo promotes SARS-CoV-2 clearance in hamsters and humans (82). Notably, IFITM3 is among the most highly induced genes in primary human lung epithelial cells exposed to SARS-CoV-2 (83, 84), and patients experiencing mild or moderate COVID-19 showed elevated induction of antiviral genes, including *IFITM1* and *IFITM3*, in airway epithelium compared with individuals with more severe COVID-19 (85). Human *IFITM3* SNPs known as rs12252 and rs34481144, which lead to IFITM3 loss of function, have been associated with severe outcomes following influenza A virus infection as well as severe COVID-19 (86, 87). These data suggest that cell-intrinsic immunity in airways plays a role in restricting virus spread and constraining systemic pathology during infection. Therefore, downmodulation of IFITM proteins by select rapalogs may contribute to the immunocompromised state that these drugs are well known to elicit in humans. This possibility warrants the close examination of the effects of different rapalog regimens on respiratory virus acquisition and disease in humans.

## Methods

Additional details can be found in the Supplemental Methods.

### Cell lines, cell culture, inhibitors, and cytokines.

HEK293T (CRL-3216) and Calu-3 (HTB-55) cells were obtained from the American Type Culture Collection (ATCC). HeLa-ACE2, HeLa-DPP4, and A549-ACE2 cell lines were produced by transducing cells with lentivirus packaging pWPI encoding ACE2 or DPP4 and selecting with blasticidin. HeLa *IFITM1–3*–KO (C5-9) cells were purchased from ATCC (CRL-3452). HeLa *TFEB*-KO cells were provided by Ramnik J. Xavier (Broad Institute of MIT and Harvard, Cambridge, Massachusetts, USA) and were described previously (88). Primary hSAECs were purchased from ATCC (PCS-301-010). The partially immortalized nasal epithelial cell line UNCNN2TS was provided by Scott H. Randell (University of North Carolina School of Medicine, Chapel Hill, North Carolina, USA). Vero E6 cells (NR-53726) were obtained from BEI Resources. Vero-TMPRSS2 cells were a gift from Shan-Lu Liu (The Ohio State University, Columbus, Ohio, USA). All cells were cultured at 37°C with 5% CO_2_ in DMEM supplemented with 10% FBS (HyClone, Cytiva), except for UNCNN2TS cells, which were cultured in EpiX Medium (Propagenix), and hSAECs, which were cultured with airway epithelial cell basal medium (ATCC, PCS-300-030) and the bronchial epithelial cell growth kit (ATCC, PCS-300-040). Primary human nasal airway epithelial cells (hNAECs) cultured at the air-liquid interface were obtained from Epithelix (EP02MP, MucilAir Pool of Donors) and cultured according to the provider’s instructions using MucilAir culture medium. Rapamycin (product no. 553211) was obtained from MilliporeSigma. Everolimus (catalog S1120), temsirolimus (catalog S1044), ridaforolimus (catalog S5003), tacrolimus (catalog S5003), and SAR405 (catalog S7682) were obtained from Selleckchem. U18666A (product no. U3633) and bafilomycin A1 (product no. SML1661) were obtained from MilliporeSigma. Type I IFN (human recombinant IFN-β_Ser17_, NR-3085) was obtained from BEI Resources.

### Virus and pseudovirus infections.

The SARS-CoV-2 isolate USA-WA1/2020 (MN985325.1) was provided by the CDC or BEI Resources (catalog NR-52281). Virus propagation was performed in Vero E6 cells. The MA SARS-CoV-2 variant MA10 (in the USA-WA1/2020 backbone) (89) was obtained from BEI Resources (catalog NR-55329). Virus propagation was performed in Vero E6 cells and subsequently in Vero-TMPRSS2 cells. The virus was sequenced to ensure the absence of tissue culture adaptations, including furin cleavage site mutations. Virus titers were calculated by plaque assay performed in Vero E6 cells as follows: serial 10-fold dilutions were added to Vero E6 monolayers in 48-well plates for 1 hour at 37°C. Cells were overlaid with 1.5% carboxymethyl cellulose (MilliporeSigma) in modified Eagle’s medium containing 3% FBS (Gibco, Thermo Fisher Scientific), 1 mM l-glutamine, 50 units/mL penicillin, and 50 μg/mL streptomycin. Three days after infection, cells were fixed in 10% formalin and stained with crystal violet to visualize and count plaques as previously described (90). Titers were calculated as PFU/mL and normalized as described in the figure legends. The HIV-based pseudovirus was produced by transfecting HEK293T cells with 12 μg pNL4-3LucR-E- and 4 μg plasmid encoding viral glycoproteins [pcDNA3.1 spike (CoV-1, CoV-2 WA1, CoV-2 Omicron/BA.1, or MERS-CoV), pMD2.G-VSV-G, or 2 μg pPol1II-HA and 2 μg pPol1II-NA] using TransIT-293 (Mirus). Virus supernatant was harvested 72 hours after transfection and filtered through 0.45 μm filters. Pseudovirus titers were determined by p24 ELISA (XpressBio), and 100 ng p24 equivalent was added to target cells and incubated for 72 hours prior to lysis with Passive Lysis Buffer (Promega). Luciferase activity was measured using the Luciferase Assay System (Promega). VSV-based pseudovirus was produced as previously described (91). In brief, HEK293T cells were seeded in a 10 cm dish and transfected with 12 μg pcDNA3.1 CoV-2 spike using Lipofectamine 2000 (Thermo Fisher Scientific). Forty-eight hours after transfection, culture medium was removed from cells, and 1 mL VSV-luc/GFP plus VSV-G (seed particles) was added. Twenty-four hours after infection, virus supernatants were collected, clarified by centrifugation at 500*g* for 5 minutes followed by filtration with a 45 μm filter, and stored. A total of 50 μL virus supernatants was added to target cells for a period of 24 hours prior to fixation with 4% paraformaldehyde (for measurements of GFP^+^ cells by flow cytometry). For infections with replication-competent SARS-CoV-2 (WA1) assessed by plaque assay, rapamycin, everolimus, temsirolimus, or ridaforolimus (20 μM) was used to pretreat cells for 4 hours, and then the drug was washed away prior to the addition of virus at an MOI of 0.1. DMSO (MilliporeSigma) was used as a vehicle control. One hour after virus addition, cells were washed once with 1× PBS and overlaid with complete medium. Supernatants were harvested 24 hours later, and titers were determined by plaque assays performed in Vero E6 cells. For infections with replication-competent SARS-CoV-2 (WA1) assessed by RT-qPCR, primary hNAECs cultured at the air-liquid interface for 30–60 days were washed 3 times with PBS and treated with 20 μM rapamycin, ridaforolimus, or an equivalent volume of DMSO for 4 hours. Then, 5 × 10^5^ PFU were added to cells for 2 hours. Afterwards, the inoculum and compound were removed, and the cells were washed 3 times with PBS. Twenty-four and 48 hours after infection, TRIzol was added to the cells, and RNA extraction and RT-qPCR were performed. For single-round infections using HIV- or VSV-based pseudovirus, rapamycin, everolimus, temsirolimus, ridaforolimus, or tacrolimus (20 μM) was used to pretreat cells for 4 hours, and the drug was maintained for the duration of infection and until harvesting of cells for a luciferase assay or flow cytometry. DMSO (MilliporeSigma) was used as a vehicle control.

### In vivo infections of hamsters and mice with SARS-CoV-2.

Male 6- to 8-week-old golden Syrian hamsters were acclimated for 11 days following receipt. The hamsters received an intraperitoneal injection of 500 μL of rapamycin (3 mg/kg; HY-10219; MedChemExpress) or ridaforolimus (3 mg/kg; HY-50908; MedChemExpress) or an equivalent amount of DMSO (*n* = 8 hamsters per group). Four hours later, the hamsters were challenged with 6 × 10^3^ PFU SARS-CoV-2 isolate USA-WA1/2020 (amplified on Calu-3 cells) through intranasal inoculation (50 μL in each nare). Half of the hamsters in each group received a second injection on day 2 after infection. Clinical observations and weights were recorded daily until post-infection day 10. According to IACUC human euthanasia criteria, hamsters were euthanized immediately if their weight loss exceeded 20% or if agonal breathing was detected. Otherwise, hamsters were euthanized on post-infection day 10. Oral swabs were collected on day 2 after infection for measurement of viral RNA by RT-qPCR of the viral N (nucleocapsid) gene. Lungs were harvested following euthanasia (day 10 or earlier), and the infectious viral load was determined by median tissue culture infectious dose (TCID_50_) assay in Vero-TMPRSS2 cells. Histopathologic analysis of hamster lungs was performed by Experimental Pathology Laboratories Inc. At necropsy, the left lung lobe was collected, placed in 10% neutral buffered formalin, and processed onto H&E-stained slides and examined by a board-certified pathologist. Histopathologic findings are presented in Supplemental Figure 8C, Supplemental Table 1, and Supplemental Data. Findings were graded from 1 to 5 (increasing severity). Male C57BL/6 mice received an intraperitoneal injection of 3 mg/kg rapamycin (NC9362949; LC-Laboratories) or an equivalent amount of DMSO (*n* = 7 mice and *n* = 6 mice per group, respectively). The following day, mice were challenged intranasally with a 5 × 10^4^ TCID_50_ equivalent dose of MA10 SARS-CoV-2 (USA-WA1/2020 backbone). Mice received a second injection of rapamycin or DMSO on the day of infection and a third on day 1 after infection. Mice were euthanized for lung harvesting on post-infection day 2. The infectious viral load was determined by TCID_50_ assay in Vero-TMPRSS2 cells. Following UV inactivation of lung homogenates, IL-6 protein was detected by Hamster IL-6 Sandwich ELISA Kit (AssayGenie) or a Mouse IL-6 Duoset Sandwich ELISA Kit (R&D Systems) according to the manufacturers’ instructions.

### Study approval.

Animal studies were conducted in compliance with all relevant local, state, and federal regulations and were approved by the IACUCs of Bioqual and The Ohio State University.

## Author contributions

AAC and GS designed the research studies and wrote the manuscript. GS, AIC, TL, ADK, SM, KKL, TD, AZ, ACE, LZ, and SK conducted experiments and acquired and analyzed data. PAB provided reagents. JWY, SMB, JSY, and AAC obtained funding and supervised the experiments. All authors contributed to editing of the manuscript.

## Supplementary Material

Supplemental data

## Version 1 10/20/2022

In-Press Preview

## Version 2 12/15/2022

Electronic publication
