# Supplementary material for: Rapalogs downmodulate intrinsic immunity and promote cell entry of SARS-CoV-2
Source: J Clin Invest. 2022 Dec 15;132(24):e160766. doi: 10.1172/JCI160766 (PMC9753997; doi:10.1172/JCI160766)
Supplement: Supplemental data [file jci-132-160766-s083.pdf]

Supplemental Figure 1

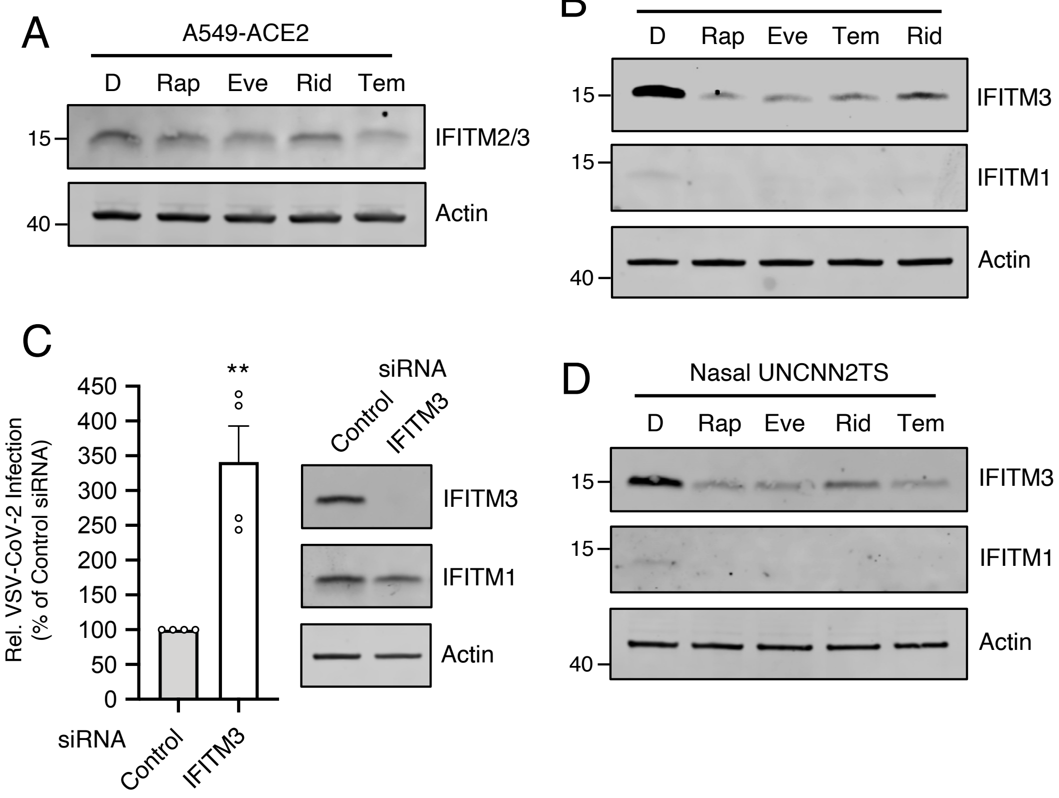

Supplemental Figure 1: (A) A549-ACE2 cells were treated with 20 µM Rap, Eve, Rid, Tem, or an equivalent volume of DMSO (in the absence of type-I interferon) for 4 hours and whole cell lysates were subjected to SDS-PAGE and Western blot analysis. Immunoblotting was performed with anti-IFITM2/3 and anti-actin. (B) Primary HSAEC were treated with 20 µM Rap, Eve, Tem, Rid, or an equivalent volume of DMSO for 4 hours and whole cell lysates were subjected to SDS-PAGE and Western blot analysis. Immunoblotting was performed with anti-IFITM2 (not detected), anti-IFITM3, anti-IFITM1, and anti-actin. (C) Primary HSAEC were transfected with siRNA targeting IFITM3 or control siRNA for 48 hours. VSV-CoV-2 (50 µL) was added to cells and infection was measured by GFP expression at 24 hours post-infection using flow cytometry. siRNA-transfected cells were subjected to SDS-PAGE and Western blot analysis. Immunoblotting was performed with anti-IFITM2 (not detected), anti-IFITM3, anti-IFITM1, and anti-actin. (D) Semi-transformed nasal epithelial cells (UNCNN2TS) were treated with 20 µM Rap, Eve, Tem, Rid, or an equivalent volume of DMSO for 4 hours and whole cell lysates were subjected to SDS-PAGE and Western blot analysis. Immunoblotting was performed with anti-IFITM2 (not detected), anti-IFITM3, anti-IFITM1, and anti-actin. Immunoblots are representative of 3 independent experiments. Means and standard error were calculated from 4 experiments. Statistical analysis was performed with student's T test and asterisks indicate significant difference from control siRNA. *, $p < 0.05$; **, $p < 0.01$. Rel.; relative.

Supplemental Figure 2

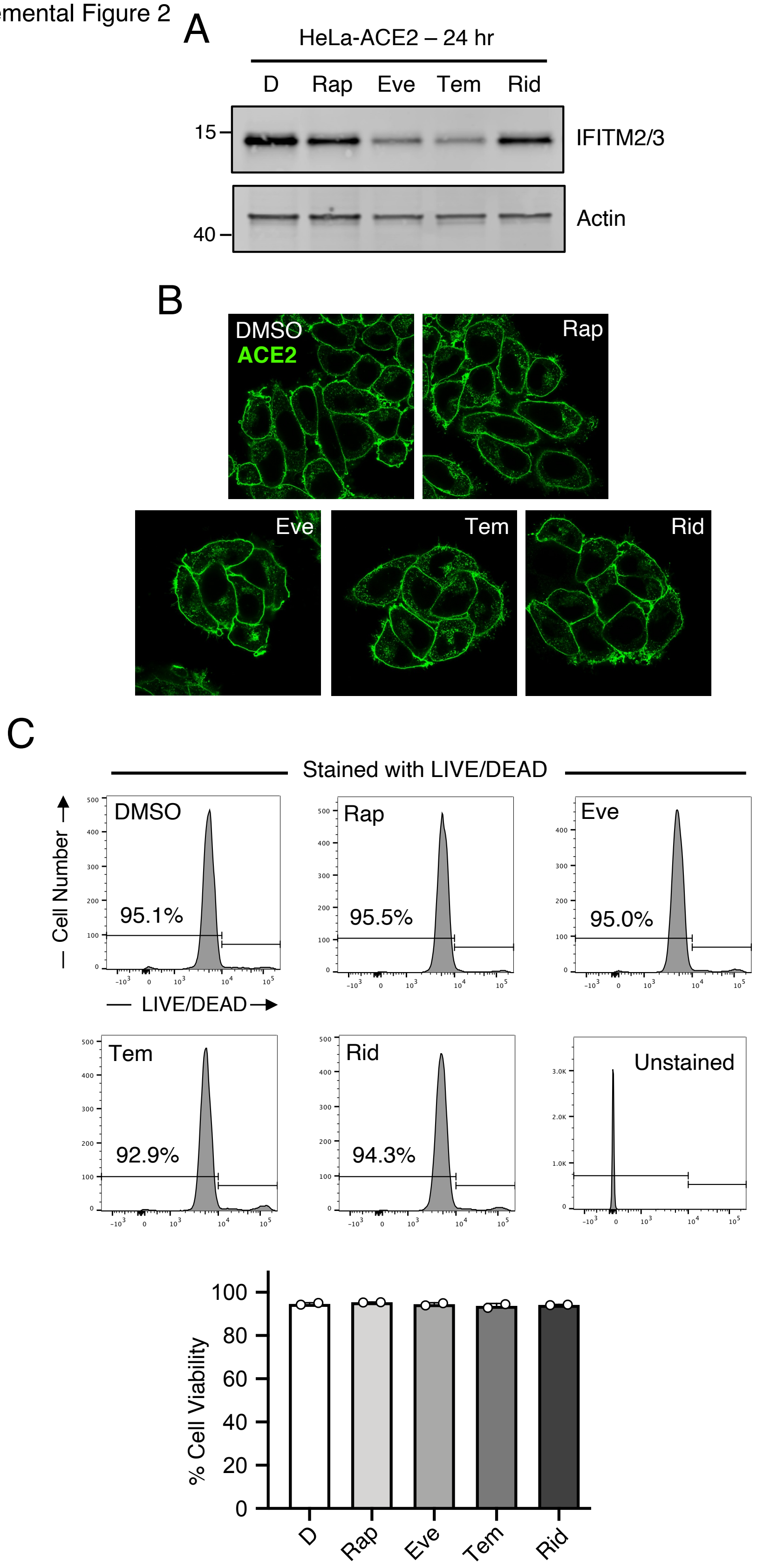

Supplemental Figure 2: (A) HeLa-ACE2 were treated with 20 μM Rap, Eve, Tem, Rid, or an equivalent volume of DMSO for 24 hours and whole cell lysates were subjected to SDS-PAGE and Western blot analysis. Immunoblotting was performed with anti-IFITM2/3 and anti-actin. (B) HeLa-ACE2 cells were treated with 20 μM Rap, Eve, Tem, Rid, or the equivalent volume of DMSO for 4 hours and cells were fixed, permeabilized, stained with anti-ACE2, and imaged by confocal immunofluorescence microscopy. Images represent a single, medial Z section. (C) HeLa-ACE2 cells were treated with 20 μM Rap, Eve, Tem, Rid, or the equivalent volume of DMSO for 4 hours and subsequently fixed and stained with LIVE/DEAD Fixable Red Dead Cell Stain Kit for 30 minutes according to manufacturer's instructions. Cells were analyzed by flow cytometry. Means and standard error were calculated from 2 experiments. All immunoblots are representative of three independent experiments.

Supplemental Figure 3

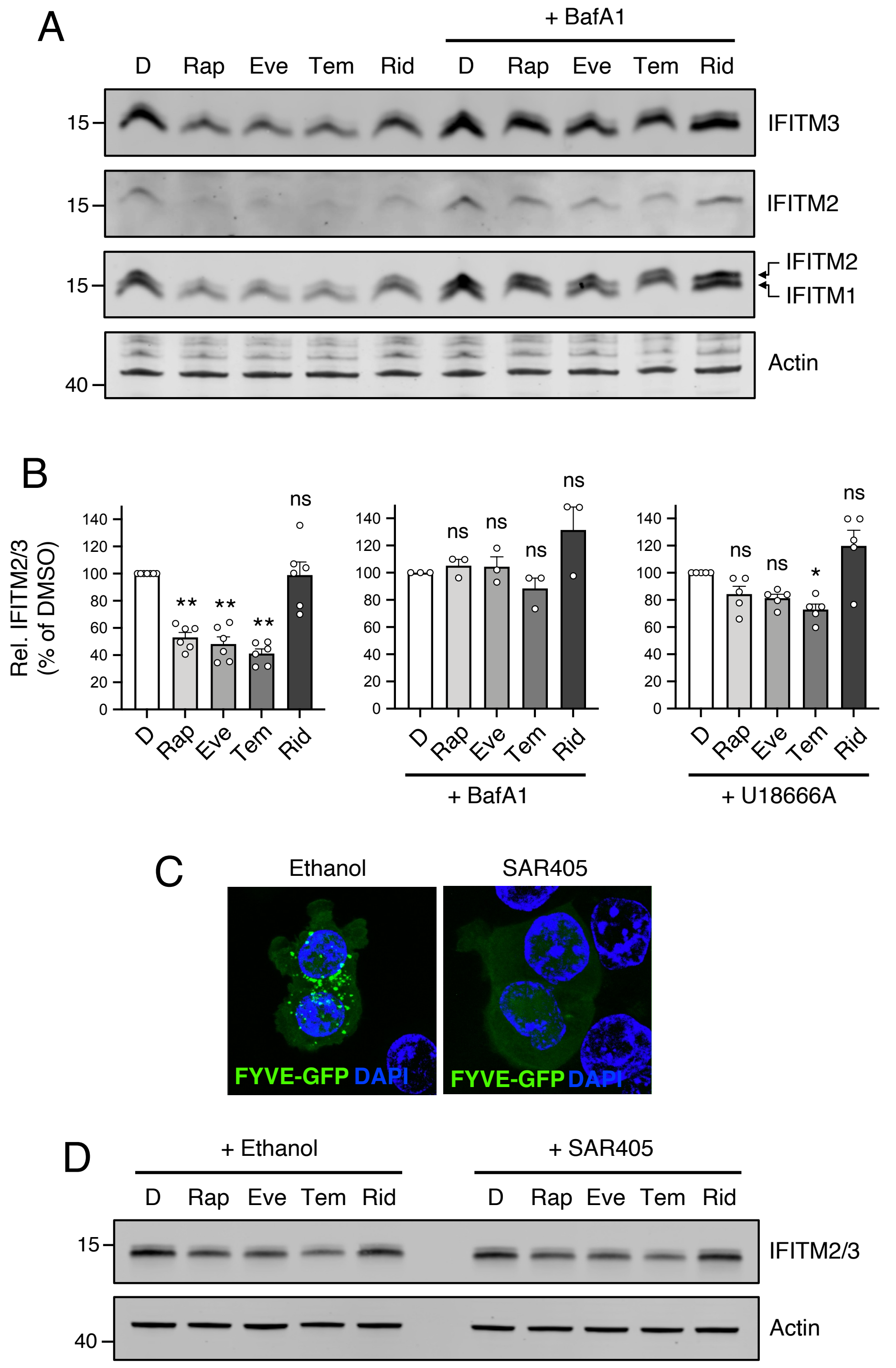

Supplemental Figure 3: (A) HeLa-ACE2 were treated with 20 μM Rap, Eve, Tem, Rid, or an equivalent volume of DMSO, in the presence or absence of 1 μM Bafilomycin A1, for 4 hours and whole cell lysates were subjected to SDS-PAGE and Western blot analysis. Immunoblotting was performed with anti-IFITM2, anti-IFITM1, anti-IFITM3, and anti-actin (in that order) on the same nitrocellulose membrane. (B) HeLa-ACE2 were treated with 20 μM Rap, Eve, Tem, Rid, or an equivalent volume of DMSO in the presence of 1 μM Bafilomycin A1, 5 μg/mL U18666A, or neither, for 4 hours. Cells were then fixed, permeabilized, and stained with anti-IFITM2/3. IFITM2/3 protein levels were measured using flow cytometry. Means and standard error were calculated from 3-6 experiments. (C) HeLa-ACE2 cells were transected with FYVE-GFP for 24 hours followed by treatment with 100 nM SAR405 or an equivalent volume of ethanol (vehicle) for 3 hours. Cells were fixed and imaged by confocal immunofluorescence microscopy. For each condition, a Z-stack of 25 slices is shown as a maximum intensity projection. (D) HeLa-ACE2 were treated with 20 μM Rap, Eve, Tem, Rid, or an equivalent volume of DMSO in the presence or absence of 100 nM SAR405 for 4 hours and whole cell lysates were subjected to SDS-PAGE and Western blot analysis. Immunoblotting was performed with anti-IFITM2/3 and anti-actin on the same nitrocellulose membrane. Statistical analysis was performed with one-way ANOVA and asterisks indicate significant difference from DMSO. *, $p < 0.05$; **, $p < 0.01$. ns; not significant. Rel.; relative.

Supplemental Figure 4

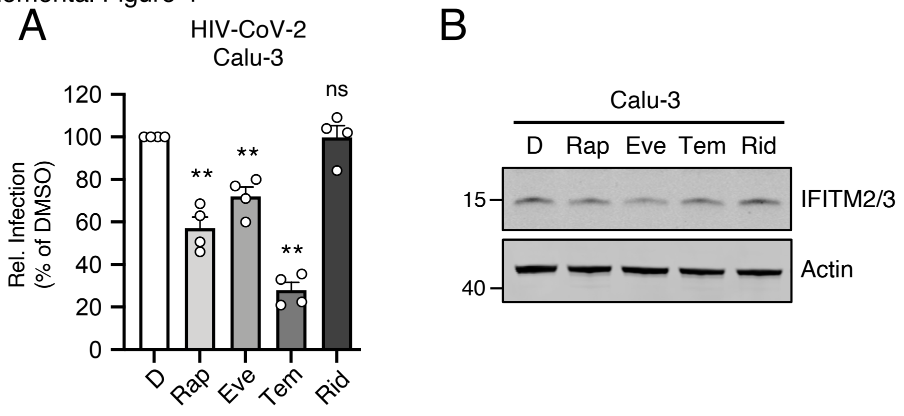

Supplemental Figure 4: (A) Calu-3 cells were treated with 20 µM Rap, Eve, Tem, Rid, or the equivalent volume of DMSO for 4 hours. HIV-CoV-2 (100 ng p24 equivalent) was added to cells and infection was measured by luciferase activity at 48 hours post-infection. Luciferase units were normalized to 100 in the DMSO condition. Means and standard error were calculated from 4 experiments. Statistical analysis was performed with one-way ANOVA and asterisks indicate significant difference from DMSO. *, $p < 0.05$; **, $p < 0.01$. ns; not significant. Rel.; relative. (B) Calu-3 cells were treated with 20 µM Rap, Eve, Tem, Rid, or the equivalent volume of DMSO for 4 hours. Whole cell lysates were subjected to SDS-PAGE and Western blot analysis. Immunoblotting was performed with anti-IFITM2/3 and anti-actin on the same nitrocellulose membrane.

Supplemental Figure 5

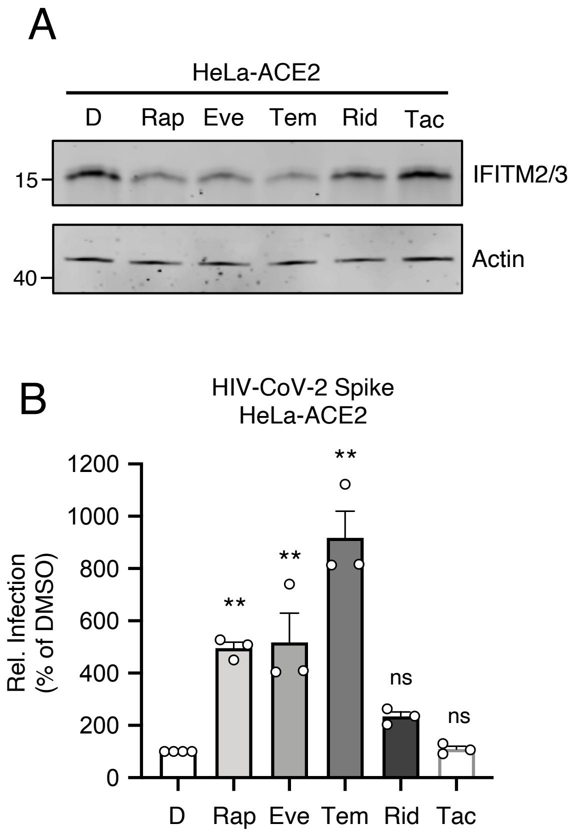

Supplemental Figure 5: (A) HeLa-ACE2 cells were treated with 20 µM Rap, Eve, Tem, Rid, Tac, or the equivalent volume of DMSO for 4 hours. Whole cell lysates were subjected to SDS-PAGE and Western blot analysis. Immunoblotting was performed with anti-IFITM2/3 and anti-actin on the same nitrocellulose membrane. (B) HeLa-ACE2 cells were treated with 20 µM Rap, Eve, Tem, Rid, Tac, or the equivalent volume of DMSO for 4 hours. HIV-CoV-2 (100 ng p24 equivalent) was added to cells and infection was measured by luciferase activity at 48 hours post-infection. Luciferase units were normalized to 100 in the DMSO condition. Means and standard error were calculated from 3 experiments. Statistical analysis was performed with one-way ANOVA and asterisks indicate significant difference from DMSO. *, $p < 0.05$; **, $p < 0.01$. ns; not significant. Rel.; relative.

Supplemental Figure 6

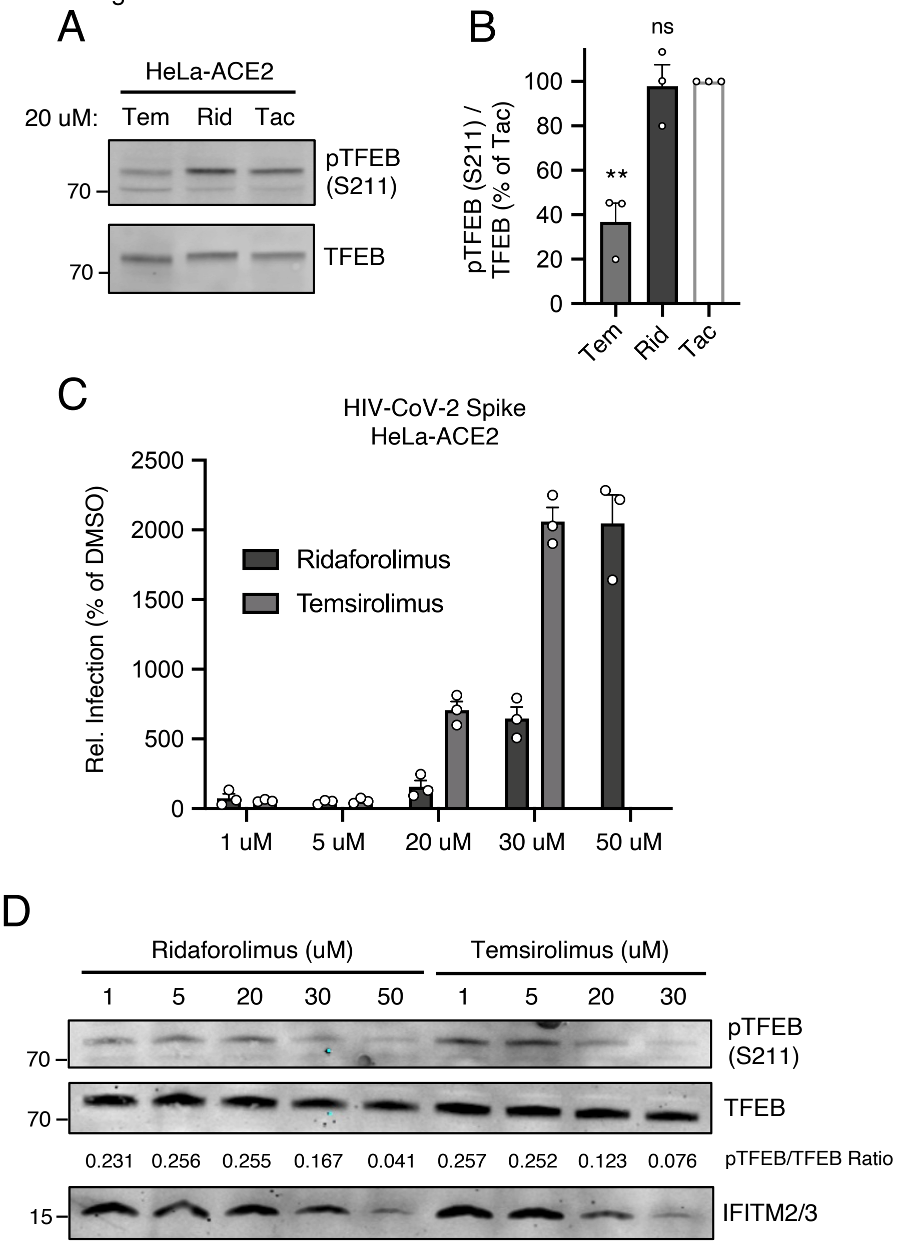

Supplemental Figure 6: (A) HeLa-ACE2 cells were treated with 20 µM Tem, Rid, or Tac for 4 hours. Whole cell lysates were subjected to SDS-PAGE and Western blot analysis. Immunoblotting was performed with anti-pTFEB (S211) and anti-TFEB on the same nitrocellulose membrane. (B) pTFEB (S211) levels were divided by total TFEB levels and summarized as an average of 3 experiments. (C) HeLa-ACE2 cells were treated with the indicated concentrations of Tem or Rid for 4 hours. HIV-CoV-2 (100 ng p24 equivalent) was added to cells and infection was measured by luciferase activity at 48 hours post-infection. Luciferase units were normalized to 100 in the DMSO condition. Means and standard error were calculated from 3 experiments. Rel.; relative. (D) HeLa-ACE2 cells were treated with the indicated concentrations of Tem or Rid for 4 hours and subjected to SDS-PAGE and Western blot analysis. Immunoblotting was performed

with anti-pTFEB (S211), anti-TFEB, and anti-IFITM2/3 on the same nitrocellulose membrane. Levels of pTFEB (S211) were divided by total TFEB to obtain pTFEB/TFEB ratios.

Supplemental Figure 7

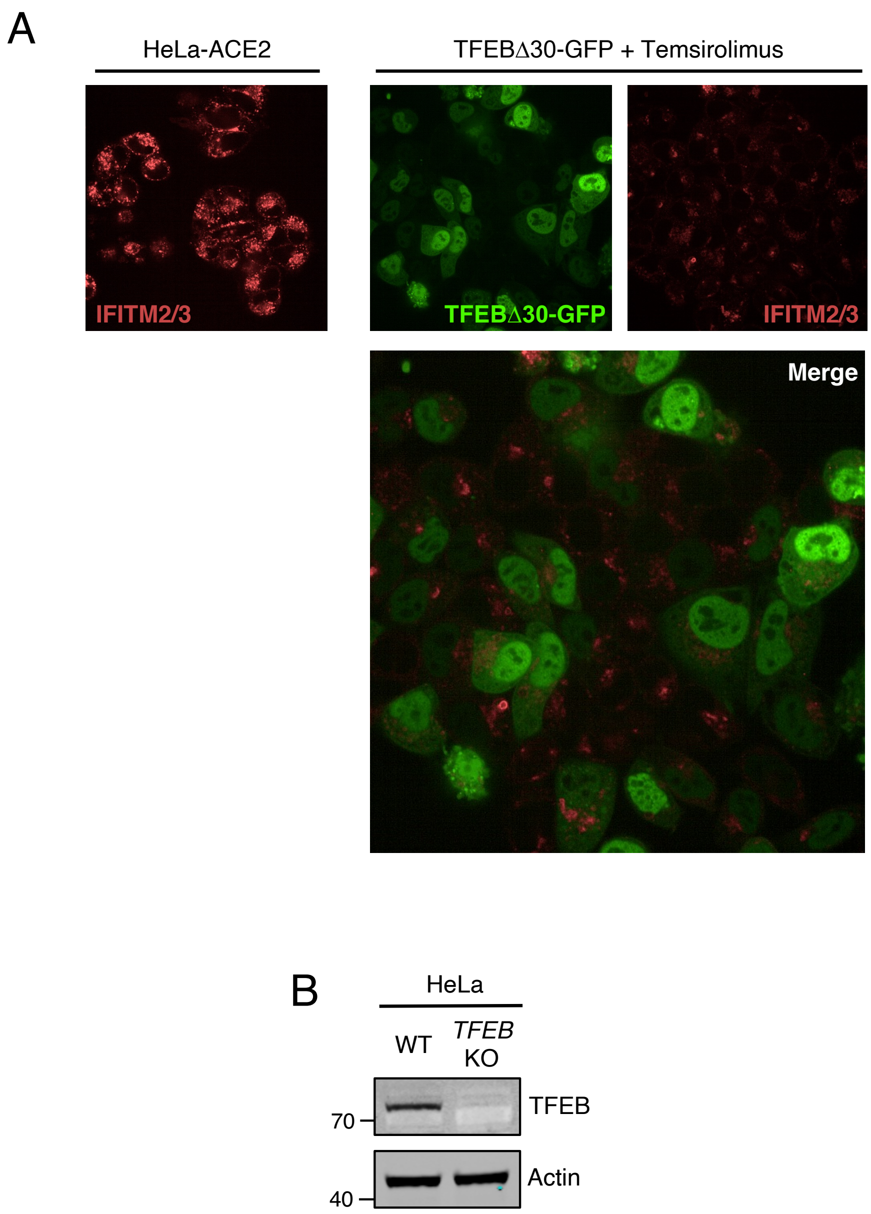

Supplemental Figure 7: (A) HeLa-ACE2 were transfected with 0.5 µg TFEBΔ30-GFP for 24 hours and treated with 20 µM Tem for four hours. Cells were then fixed, permeabilized, stained with anti-IFITM2/3, and imaged by confocal immunofluorescence microscopy. Representative images are shown and anti-IFITM2/3 staining in untreated HeLa-ACE2 are shown for comparison. (B) Whole cell lysates from HeLa WT and HeLa *TFEB* KO cells were subjected to SDS-PAGE and Western blot analysis. Immunoblotting was performed with anti-TFEB and anti-actin on the same nitrocellulose membrane.

Supplemental Figure 8

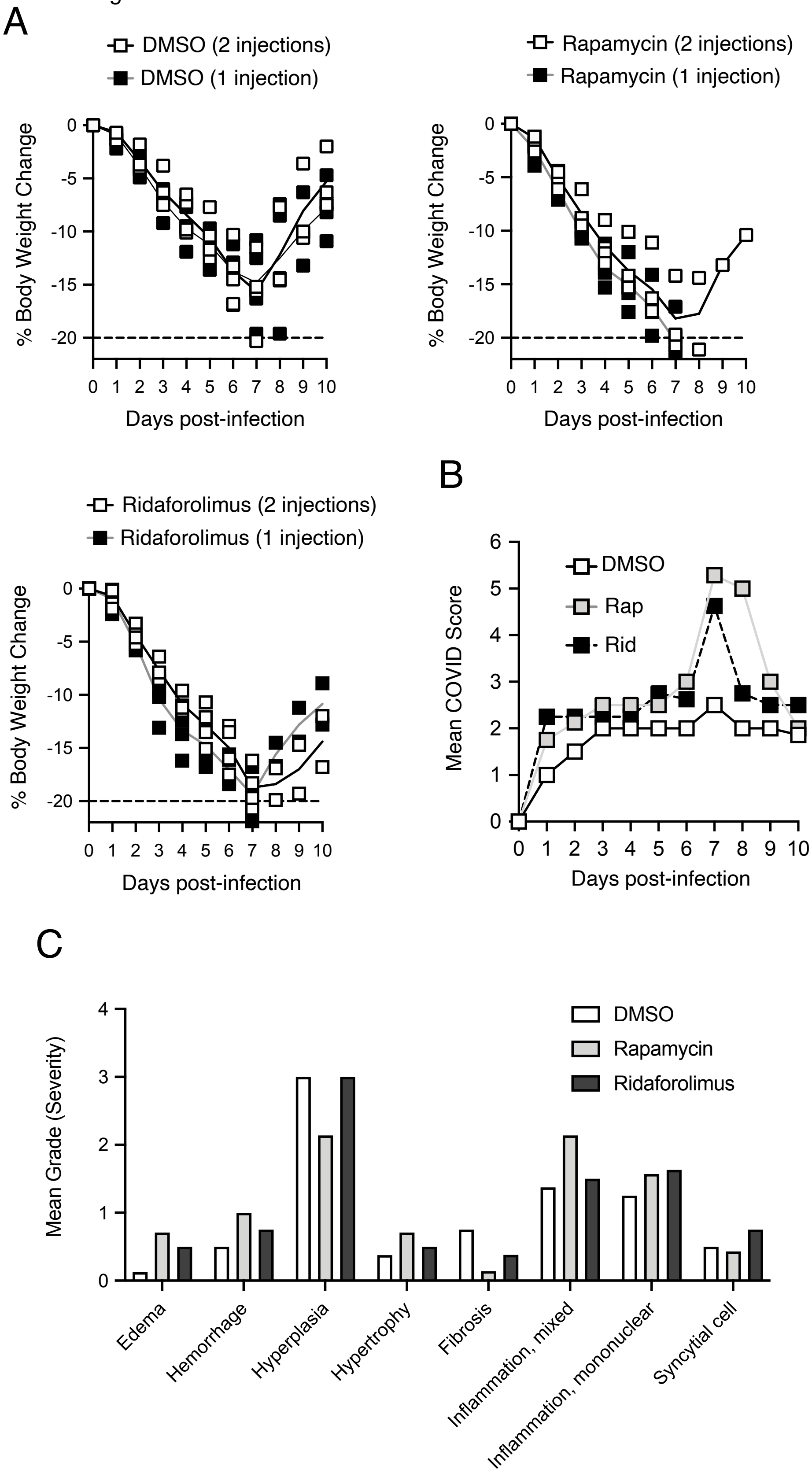

Supplemental Figure 8: (A) Body weight measurements for individual hamsters following injections with DMSO, Rap, or Rid are plotted by day post-infection and presented as % body weight change relative to Day 0. Hamsters receiving one injection of 3 mg/kg DMSO, Rap, or Rid prior to infection (n=4, 1 injection) are indicated by black squares, while hamsters receiving one injection prior to infection as well as a second injection of 3 mg/kg DMSO, Rap, or Rid at Day 2 post-infection (n=4, 2 injections) are indicated by white squares. The average daily weight change for each group is indicated by grey and black lines, respectively. If and when a hamster lost 20% or more of its body weight or exhibited agonal breathing, it was euthanized and body weight measurements were stopped. (B) Mean COVID scores were calculated for infected hamsters treated with DMSO, Rap, or Rid. Each hamster was observed daily and scores were calculated additively as follows: mild ruffling of fur (1 point), hunched posture (1 point), moderate/severe ruffling of fur (2 points), lethargic (3 points), more than 20% weight loss (4 points). (C) Results of microscopic histopathology of infected hamsters are presented as mean grade (severity) for each treatment group. Grades ranged from 1 to 4 (low to high severity).

Supplemental Figure 9

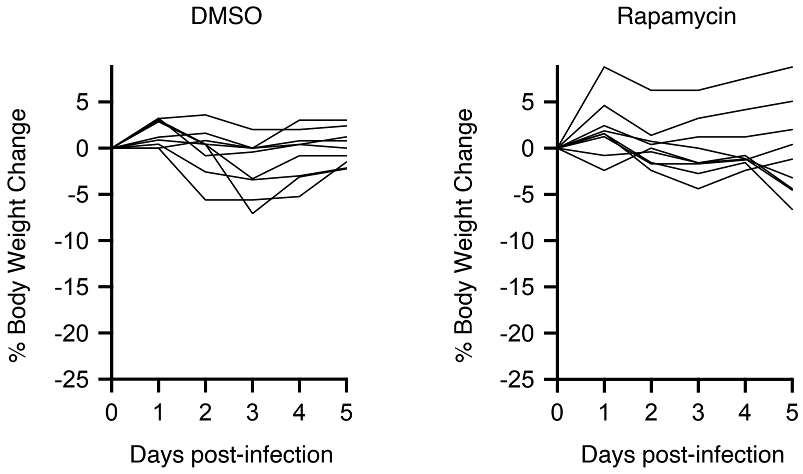

Supplemental Figure 9: Body weight trajectories for individual C57BL/6 mice treated with DMSO (n = 9) or Rap (n = 9) and infected with MA-SARS-CoV-2 are plotted by day post-infection (up to 5 days).

Supplemental Table 1: Overview of hamster lung pathology

Group	ID	Number of injections	Day of euthanasia	Basis for euthanasia	Gross lung observations	COVID score on day of euthanasia	Lung $TCID_{50}$ (per gram)	Lung IL-6 (pg/mL)
DMSO	2527	2 (pre-, post-infection)	D10	End of Expt.	Abnormal	2	BD	168.7
	2528		D7	Morbid (WL)	Severely hemorrhagic	6	1,879,699	69.3
	2529		D10	End of Expt.	Abnormal	2	BD	123.8
	2530		D10	End of Expt.	Abnormal	2	BD	45.7
	2539	1 (pre-infection)	D10	End of Expt.	Abnormal	2	BD	168.7
	2540		D10	End of Expt.	Abnormal	1	BD	136.6
	2541		D10	End of Expt.	Abnormal	2	BD	162.4
	2542		D10	End of Expt.	Abnormal	2	BD	113.7
Rap.	2531	2 (pre-, post-infection)	D6	Morbid (AB)	Moderately hemorrhagic	7	3,340,565	51.5
	2532		D7	Morbid (WL)	Severely hemorrhagic	7	1,479,290	49.6
	2533		D8	Morbid (WL)	Severely hemorrhagic	7	49,104	56.9
	2534		D10	End of Expt.	Abnormal	2	BD	56.9
	2543	1 (pre-infection)	D7	Found Dead	N/A	6	N/A	N/A
	2544		D7	Morbid (WL)	Severely hemorrhagic	6	46,232	66.8
	2545		D7	Morbid (WL)	Severely hemorrhagic	6	585,607	36.3
	2546		D8	Morbid (WL)	Severely hemorrhagic	6	1,196,837	53.4
Rid.	2535	2 (pre-, post-infection)	D10	End of Expt.	Abnormal	3	BD	107.8
	2536		D8	Morbid (AB)	Moderately hemorrhagic	6	201,613	36.1
	2537		D8	Morbid (WL)	Severely hemorrhagic	6	52,705	67.0
	2538		D10	End of Expt.	Abnormal	2	BD	97.6
	2547	1 (pre-infection)	D7	Morbid (WL)	Severely hemorrhagic	7	47,722	69.5
	2548		D7	Morbid (WL)	Severely hemorrhagic	7	29,899	63.0
	2549		D10	End of Expt.	Abnormal	2	BD	172.8
	2550		D10	End of Expt.	Abnormal	3	BD	202.2

WL; weight loss. AB; agonal breathing. BD; below detection (1,667)

Supplemental Methods

Plasmids and RNA interference

pcDNA3.1 encoding human ACE2 was kindly provided by Thomas Gallagher (Loyola University). pcDNA3.1 encoding CoV-1 Spike or CoV-2 Spike (WA1) tagged with a C9 epitope on the C-terminus, or MERS Spike, was kindly provided by Thomas Gallagher (Loyola University). pcDNA3.1 encoding CoV-1 Spike or CoV-2 Spike (WA1) tagged with a FLAG epitope on the C-terminus was obtained from Michael Letko and Vincent Munster (NIAID). pcDNA3.1 encoding CoV-2 Omicron (BA.1) Spike tagged with a His epitope on the N-terminus was synthesized provided by Genscript. pMD2.G encoding VSV-G (12259) was obtained from Addgene (a generous gift from Didier Trono). pWPI was obtained from Addgene (12254) and human ACE2 or human TMPRSS2 was introduced by Gateway cloning (Gateway LR Clonase II Enzyme mix (11791020)) as per manufacturer's instructions. pPolII encoding hemagglutinin (HA) or neuraminidase (NA) from Influenza A/Turkey/1/2005 (H5N1) were kindly provided by Richard Yi Tsun Kao (The University of Hong Kong). pCMV encoding HIV-1 Vpr fused to beta lactamase (pCMV4-BlaM-Vpr) was obtained from Addgene (21950). A plasmid encoding replication-incompetent HIV-1 lacking *env* and *vpr* and encoding luciferase (pNL4-3LucR-E-) was kindly provided by Vineet KewalRamani (National Cancer Institute). A plasmid encoding replication-incompetent HIV-1 lacking *env* (pNL4-3E-) was kindly provided by Olivier Schwartz (Institut Pasteur). pEGFP-N1-TFEB (38119) and pEGF-N1-Δ30TFEB (44445) were obtained from Addgene (a generous gift of Shawn M. Ferguson). pEGFP-2xFYVE (140047) was obtained from Addgene (a gift from Harald Stenmark). Silencer Select siRNA targeting IFITM3 (s195035) and a non-targeting control (No. 1) was obtained from Ambion. Cells were transfected with 20 nM siRNA using Opti-MEM (Gibco) and Lipofectamine RNAiMAX (Thermo Fisher).

FRET-based virus entry assay

HIV-based pseudovirus incorporating BlaM-Vpr and CoV-2 Spike was produced by transfecting HEK293T cells with pNL4-3E- (15 μg), pCMV4-BlaM-Vpr (5 μg), and pcDNA3.1 CoV-2 Spike (5 μg) using the calcium phosphate technique. Briefly, six million 293T cells were seeded in a T75 flask. Plasmid DNA was mixed with sterile H_2O, $CaCl_2$, and Tris-EDTA (TE) buffer, and the totality was combined with Hepes-buffered saline (HBS). The transfection volume was added dropwise, and cells were incubated at 37°C for 48 h. Supernatants were recovered and clarified by centrifugation, passed through a 0.45 μm filter, and stored. Titers were measured using an HIV-1 p24 ELISA kit (XpressBio). 50 ng p25 equivalent of virus was added to HeLa-ACE2 cells for 2 hours. Cells were washed and labeled with the CCF2-AM β-lactamase Loading Kit (Invitrogen) for 2 hours and analyzed for CCF2 cleavage by flow cytometry as described (1). Rapamycin, everolimus, temsirolimus, or ridaforolimus (20 μM) were used to pretreat cells for 4 hours prior to virus addition and were maintained for the duration of infection. DMSO (Sigma) was used as a vehicle control.

Western blot, antibodies, and flow cytometry

Whole cell lysis was performed with RIPA buffer (Thermo Fisher) supplemented with Halt Protease Inhibitor EDTA-free (Thermo Fisher). Lysates were clarified by centrifugation and

supernatants were collected and stored. Protein concentration was determined with the Protein Assay Kit II (Bio-Rad), and 10-15 μg of protein was loaded into 12% acrylamide Criterion XT Bis-Tris Precast Gels (Bio-Rad). Electrophoresis was performed with NuPage MES SDS Running Buffer (Invitrogen) and proteins were transferred to Amersham Protran Premium Nitrocellulose Membrane, pore size 0.20 μm (GE Healthcare). Membranes were blocked with Odyssey Blocking Buffer (Li-COR) and incubated with the following primary antibodies diluted in Odyssey Antibody Diluent (Li-COR): anti-IFITM1 (60074-1-Ig; Proteintech), anti-IFITM2 (66137-1-Ig; Proteintech), anti-IFITM3 (EPR5242, ab109429; Abcam), anti-Fragilis (ab15592; Abcam (detects murine IFITM3)), anti-IFITM2/3 (66081-1-Ig; Proteintech), anti-actin (C4, sc-47778; Santa Cruz Biotechnology), anti-hACE2 (ab15348; Abcam), anti-TFEB (4240S; Cell Signaling Technology), and anti-pTFEB (Ser211) (37681S; Cell Signaling Technology). Secondary antibodies conjugated to DyLight 800 or 680 (Li-Cor) and the Li-Cor Odyssey CLx imaging system were used to reveal specific protein detection. Images were analyzed (including signal quantification) and assembled using ImageStudioLite (Li-Cor). Cell viability was measured using LIVE/DEAD Red Dead Cell Stain Kit (Thermo Fisher). Cells were fixed and permeabilized with Cytofix/Cytoperm reagent (BD) for 20 minutes and washed in Perm/Wash buffer (BD). Flow cytometry was performed on an LSRFortessa (BD).

Confocal fluorescence and immunofluorescence microscopy

HeLa-ACE2 cells were fixed with 4% paraformaldehyde, stained with anti-IFITM2/3 (66081-1-Ig; Proteintech), goat anti-mouse IgG Alexa Fluor 647 (A21235; Thermo Fisher) and DAPI (62248; Thermo Fisher), and imaged in a glass-bottom tissue culture plate with an Operetta CLS High-Content Analysis System (Perkin Elmer). For measurement of TFEB-GFP nuclear/cytoplasmic distribution, HeLa-ACE2 cells were transfected with pEGFP-N1-TFEB for 24 hours, fixed with 4% paraformaldehyde, stained with HCS CellMask Red Stain (H32712; Thermo Fisher) and DAPI, and imaged with an Operetta CLS. Using Harmony software (Perkin Elmer), nuclear/cytoplasmic ratios of TFEB-GFP were calculated in single cells as follows: cells were delineated by CellMask Red Stain, nuclei were delineated by DAPI, nuclear TFEB-GFP was designated as GFP overlapping with DAPI, and cytoplasmic TFEB-GFP was designated as total GFP signal minus nuclear TFEB-GFP. Average ratios were calculated from 20-30 cells per field, and the mean of averages from 10 fields was obtained (total of approximately 250 cells per condition). For measurement of IFITM2/3 levels in cells transfected with TFEBΔ30-GFP, HeLa-ACE2 cells were transfected with pEGF-N1-Δ30TFEB for 24 hours, fixed and permeabilized with BD Cytofix/Cytoperm (Fisher Scientific), stained with anti-IFITM2/3 and goat anti-mouse IgG Alexa Fluor 647, and imaged with an Operetta CLS. The IFITM2/3 fluorescence intensity within a single, medial Z section was measured in approximately 150 GFP-negative cells and 150 GFP-positive cells using the freehand selections tool in ImageJ.

RT-qPCR of viral and cellular transcripts in infected primary human nasal epithelial cells

Cells lysed with Trizol were mixed with chloroform (Sigma) at a 5:1 (Trizol:chloroform) ratio. Mixed samples were mixed thoroughly and incubated at room temperature for 10 minutes, followed by centrifugation at 12000 x G for 5 minutes to allow separation of the aqueous and organic phases. Equal volumes of 70% ethanol were added to the aqueous phases, mixed thoroughly, and incubated at room temperature for 5 minutes. RNA purification was performed

using the PureLink RNA Mini Kit (Invitrogen) according to manufacturer's instructions. Purified RNA product was immediately used with the One-step PrimeScript RT-PCR Kit (Takara). Primers and probes were obtained from IDT. The primers and probes used to amplify and quantify *ORF1a* are as follows (5'-3'): ORF1a-F AGAAGATTGGTTAGATGATGATAGT; ORF1a-R TTCCATCTCTAATTGAGGTTGAACC; ORF1a-P FAM/TCCTCACTGCCGTCTTGTTGACCA/BHQ13. The primers and probes used to amplify and quantify *IL6* are as follows (5'-3'): IL6-F GCAGATGAGTACAAAAGTCCTGA; IL6-R TTCTGTGCCTGCAGCTTC; IL6-P 56-FAM/CAACCACAA/ZEN/ATGCCAGCCTGCT/31ABkFQ. The primers and probes used to amplify and quantify *IFNB1* are as follows (5'-3'): IFNB1-F GAAACTGAAGATCTCCTAGCCT; IFNB1-R GCCATCAGTCACTTAAACAGC; IFNB1-P 56-FAM/TGAAGCAAT/ZEN/TGTCCAGTCCCAGAGG/3IABkFQ. The primers and probes used to amplify and quantify *ACTB* are as follows (5'-3'): ACTB-F ACAGAGCCTCGCCTTTG; ACTB-R CCTTGCACATGCCGGAG; ACTB-P 56-FAM/TCATCCATG/ZEN/GTGAGCTGGCGG/31ABkFQ. Reaction mixtures of 20 µL (including 2.2 µL total RNA, 0.2 µM forward and reverse primers, and 0.1 µM probe) were subjected to reverse transcription (5 min at 45°C, followed by 10 s at 95°C) and 40 cycles of PCR (5 s at 95°C followed by 20 s at 60°C) in a CFX Opus 96 Real-Time PCR System (Bio-Rad). Results were analyzed by the Comparative CT Method ($\Delta\Delta C_t$ Method). RNA levels for *ORF1a*, *IL6*, and *IFNB1* in each sample were normalized to *ACTB*.

Statistics

The statistical tests performed in each figure are described in the accompanying figure legend. In general, the cutoff (alpha) for significance was 0.05 and two-tailed tests were always performed.

1. Compton AA, Bruel T, Porrot F, Mallet A, Sachse M, Euvrard M, et al. IFITM Proteins Incorporated into HIV-1 Virions Impair Viral Fusion and Spread. *Cell Host & Microbe.* 2014;16(6):736-47.

APPENDIX
Individual histopathology reports

Animal ID	Sex	Group
2527	M	DMSO - 2 injections

Death: Scheduled, Terminal Sacrifice
Day of Death: D10
Date/Time: 06 Aug 2021 8:22 AM
Specimen: Left lung
Microscopic analysis:
- Hyperplasia - bronchiolo-alveolar, grade 3
- Inflammation, mixed cell - alveolar, interstitial, grade 1
- Inflammation, mononuclear cell - vascular/perivascular, grade 2
- Syncytial cell - grade 1

Animal ID	Sex	Group
2528	M	DMSO - 2 injections

Death: Unscheduled, Moribund Sacrifice
Day of Death: D7
Date/Time: 03 Aug 2021 8:20 AM
Specimen: Left lung
Microscopic analysis:
- Edema - perivascular, grade 1
- Hemorrhage - alveolar, grade 2
- Hyperplasia - bronchiolo-alveolar, grade 3
- Hypertrophy - mesothelium, grade 1
- Inflammation, mixed cell - alveolar, interstitial, grade 2
- Inflammation, mononuclear cell - vascular/perivascular, grade 1
- Syncytial cell - grade 1

Animal ID	Sex	Group
2529	M	DMSO - 2 injections

Death: Scheduled, Terminal Sacrifice
Day of Death: D10
Date/Time: 06 Aug 2021 8:22 AM
Specimen: Left lung
Microscopic analysis:
- Fibrosis - pleura, grade 1
- Hyperplasia - bronchiolo-alveolar, grade 4
- Inflammation, mixed cell - alveolar, interstitial, grade 2
- Inflammation, mononuclear cell - vascular/perivascular, grade 2
- Syncytial cell - grade 1

Animal ID	Sex	Group
2530	M	DMSO - 2 injections

Death: Scheduled, Terminal Sacrifice
Day of Death: D10
Date/Time: 06 Aug 2021 8:22 AM
Specimen: Left lung
Microscopic analysis:
- Fibrosis - pleura, grade 2
- Hyperplasia - bronchiolo-alveolar, grade 3
- Inflammation, mixed cell - alveolar, interstitial, grade 2
- Syncytial cell - grade 1

Animal ID	Sex	Group
2531	M	Rapamycin - 2 injections

Death: Unscheduled, Moribund Sacrifice
Day of Death: D7
Date/Time: 03 Aug 2021 8:49 AM
Specimen: Left lung
Microscopic analysis:
- Hemorrhage - alveolar, grade 1
- Hyperplasia - bronchiolo-alveolar, grade 2
- Inflammation, mixed cell - alveolar, interstitial, grade 3
- Syncytial cell - grade 1

Animal ID	Sex	Group
2532	M	Rapamycin - 2 injections

Death: Unscheduled, Moribund Sacrifice
Day of Death: D7
Date/Time: 03 Aug 2021 8:21 AM
Specimen: Left lung
Microscopic analysis:
- Edema - perivascular, grade 1
- Hemorrhage - alveolar, grade 2
- Hyperplasia - bronchiolo-alveolar, grade 2
- Hypertrophy - mesothelium, grade 1
- Inflammation, mixed cell - bronchoalveolar, interstitial, grade 3

Animal ID	Sex	Group
2533	M	Rapamycin - 2 injections

Death: Unscheduled, Moribund Sacrifice
Day of Death: D8
Date/Time: 04 Aug 2021 8:21 AM
Specimen: Left lung
Microscopic analysis:
- Edema - perivascular, grade 1
- Hyperplasia - bronchiolo-alveolar, grade 2
- Hypertrophy - mesothelium, grade 1
- Inflammation, mononuclear cell - vascular/perivascular, grade 1
- Inflammation, mononuclear cell - alveolar, interstitial, grade 3

Animal ID	Sex	Group
2534	M	Rapamycin - 2 injections

Death: Scheduled, Terminal Sacrifice
Day of Death: D10
Date/Time: 06 Aug 2021 8:22 AM
Specimen: Left lung
Microscopic analysis:
- Edema - perivascular, grade 1
- Fibrosis - pleura, grade 1
- Hyperplasia - bronchiolo-alveolar, grade 2
- Hypertrophy - mesothelium, grade 1
- Inflammation, mononuclear cell - vascular/perivascular, grade 1
- Inflammation, mononuclear cell - alveolar, interstitial, grade 3

**Animal ID**	**Sex**	**Group**
2535	M	Ridaforolimus - 2 injections

Death: Scheduled, Terminal Sacrifice
Day of Death: D10
Date/Time: 06 Aug 2021 8:22 AM
Specimen: Left lung
Microscopic analysis:
Hyperplasia - bronchiolo-alveolar, grade 2
Inflammation, mononuclear cell - alveolar, interstitial, grade 2

**Animal ID**	**Sex**	**Group**
2536	M	Ridaforolimus - 2 injections

Death: Unscheduled, Moribund Sacrifice
Day of Death: D8
Date/Time: 04 Aug 2021 8:21 AM
Specimen: Left lung
Microscopic analysis:
Edema - perivascular, grade 1
Fibrosis - mesothelium, grade 1
Hemorrhage - alveolar, grade 2
Hyperplasia - bronchiolo-alveolar, grade 3
Inflammation, mixed cell - alveolar, interstitial, grade 3
Inflammation, mononuclear cell - vascular/perivascular, grade 1
Syncytial cell - grade 1

**Animal ID**	**Sex**	**Group**
2537	M	Ridaforolimus - 2 injections

Death: Unscheduled, Moribund Sacrifice
Day of Death: D7
Date/Time: 03 Aug 2021 8:21 AM
Specimen: Left lung
Microscopic analysis:
Edema - perivascular, grade 1
Hemorrhage - alveolar, grade 1
Hyperplasia - bronchiolo-alveolar, grade 3
Hypertrophy - mesothelium, grade 2
Inflammation, mixed cell - bronchoalveolar, interstitial, grade 3
Inflammation, mononuclear cell - vascular/perivascular, grade 1
Syncytial cell - grade 1

**Animal ID**	**Sex**	**Group**
2538	M	Ridaforolimus - 2 injections

Death: Scheduled, Terminal Sacrifice
Day of Death: D10
Date/Time: 06 Aug 2021 8:22 AM
Specimen: Left lung
Microscopic analysis:
Hyperplasia - bronchiolo-alveolar, grade 4
Inflammation, mononuclear cell - vascular/perivascular, grade 1
Inflammation, mononuclear cell - alveolar, interstitial, grade 2
Syncytial cell - grade 1

**Animal ID**	**Sex**	**Group**
2539	M	DMSO - 1 injection

Death: Scheduled, Terminal Sacrifice
Day of Death: D10
Date/Time: 06 Aug 2021 8:22 AM
Specimen: Left lung
Microscopic analysis:
Fibrosis - pleura, grade 1
Hemorrhage - alveolar, grade 1
Hyperplasia - bronchiolo-alveolar, grade 3
Hypertrophy - mesothelium, grade 1
Inflammation, mixed cell - alveolar, interstitial, grade 1
Inflammation, mononuclear cell - vascular/perivascular, grade 1

**Animal ID**	**Sex**	**Group**
2540	M	DMSO - 1 injection

Death: Scheduled, Terminal Sacrifice
Day of Death: D10
Date/Time: 06 Aug 2021 8:22 AM
Specimen: Left lung
Microscopic analysis:
Hyperplasia - bronchiolo-alveolar, grade 2
Inflammation, mononuclear cell - vascular/perivascular, grade 1
Inflammation, mononuclear cell - alveolar, interstitial, grade 2

**Animal ID**	**Sex**	**Group**
2541	M	DMSO - 1 injection

Death: Scheduled, Terminal Sacrifice
Day of Death: D10
Date/Time: 06 Aug 2021 8:22 AM
Specimen: Left lung
Microscopic analysis:
Fibrosis - pleura, grade 1
Hemorrhage - alveolar, grade 1
Hyperplasia - bronchiolo-alveolar, grade 3
Hypertrophy - mesothelium, grade 1
Inflammation, mixed cell - alveolar, interstitial, grade 2
Inflammation, mononuclear cell - vascular/perivascular, grade 1

**Animal ID**	**Sex**	**Group**
2542	M	DMSO - 1 injection

Death: Scheduled, Terminal Sacrifice
Day of Death: D10
Date/Time: 06 Aug 2021 8:22 AM
Specimen: Left lung
Microscopic analysis:
Fibrosis - pleura, grade 1
Hyperplasia - bronchiolo-alveolar, grade 3
Inflammation, mononuclear cell - alveolar, interstitial, grade 1

Animal ID	Sex	Group
2543	M	Rapamycin - 1 injection

Found dead on 04 Aug 2021 - no lung pathology performed

Animal ID	Sex	Group
2544	M	Rapamycin - 1 injection

Death: Unscheduled, Moribund Sacrifice
Day of Death: D7
Date/Time: 03 Aug 2021 8:22 AM
Specimen: Left lung
Microscopic analysis:
- Edema - perivascular, grade 1
- Hemorrhage - alveolar, grade 1
- Hyperplasia - bronchiolo-alveolar, grade 3
- Hypertrophy - mesothelium, grade 1
- Inflammation, mixed cell - bronchoalveolar, interstitial, grade 3
- Inflammation, mononuclear cell - vascular/perivascular, grade 1

Animal ID	Sex	Group
2545	M	Rapamycin - 1 injection

Death: Unscheduled, Moribund Sacrifice
Day of Death: D7
Date/Time: 03 Aug 2021 8:22 AM
Specimen: Left lung
Microscopic analysis:
- Edema - perivascular, grade 1
- Hemorrhage - alveolar, grade 1
- Hyperplasia - bronchiolo-alveolar, grade 2
- Inflammation, mixed cell - bronchoalveolar, interstitial, grade 3
- Inflammation, mononuclear cell - vascular/perivascular, grade 2
- Syncytial cell - grade 1

Animal ID	Sex	Group
2546	M	Rapamycin - 1 injection

Death: Unscheduled, Moribund Sacrifice
Day of Death: D7
Date/Time: 03 Aug 2021 8:22 AM
Specimen: Left lung
Microscopic analysis:
- Hemorrhage - alveolar, grade 2
- Hyperplasia - bronchiolo-alveolar, grade 2
- Hypertrophy - mesothelium, grade 1
- Inflammation, mixed cell - bronchoalveolar, interstitial, grade 3
- Inflammation, mononuclear cell - vascular/perivascular, grade 2
- Syncytial cell - grade 1

Animal ID	Sex	Group
2547	M	Ridaforolimus - 1 injection

Death: Unscheduled, Moribund Sacrifice
Day of Death: D7
Date/Time: 03 Aug 2021 8:22 AM
Specimen: Left lung
Microscopic analysis:
- Edema - perivascular, grade 1
- Hemorrhage - alveolar, grade 1
- Hyperplasia - bronchiolo-alveolar, grade 3
- Hypertrophy - mesothelium, grade 1
- Inflammation, mixed cell - alveolar, interstitial, grade 3
- Inflammation, mononuclear cell - vascular/perivascular, grade 2
- Syncytial cell - grade 1

Animal ID	Sex	Group
2548	M	Ridaforolimus - 1 injection

Death: Unscheduled, Moribund Sacrifice
Day of Death: D7
Date/Time: 03 Aug 2021 8:22 AM
Specimen: Left lung
Microscopic analysis:
- Edema - perivascular, grade 1
- Hemorrhage - alveolar, grade 2
- Hyperplasia - bronchiolo-alveolar, grade 3
- Hypertrophy - mesothelium, grade 1
- Inflammation, mixed cell - alveolar, interstitial, grade 3
- Inflammation, mononuclear cell - vascular/perivascular, grade 1
- Syncytial cell - grade 1

Animal ID	Sex	Group
2549	M	Ridaforolimus - 1 injection

Death: Scheduled, Terminal Sacrifice
Day of Death: D10
Date/Time: 06 Aug 2021 8:22 AM
Specimen: Left lung
Microscopic analysis:
- Fibrosis - pleura, grade 2
- Hyperplasia - bronchiolo-alveolar, grade 3
- Inflammation, mononuclear cell - alveolar, interstitial, grade 2
- Syncytial cell - grade 1

Animal ID	Sex	Group
2550	M	Ridaforolimus - 1 injection

Death: Scheduled, Terminal Sacrifice
Day of Death: D10
Date/Time: 06 Aug 2021 8:22 AM
Specimen: Left lung
Microscopic analysis:
- Hyperplasia - bronchiolo-alveolar, grade 3
- Inflammation, mononuclear cell - vascular/perivascular, grade 2
- Inflammation, mononuclear cell - alveolar, interstitial, grade 2
